# Influence of polyfunctional Tbet^+^ T cells on specific clinical events in chronic lymphocytic leukaemia

**DOI:** 10.3389/fimmu.2025.1528405

**Published:** 2025-04-17

**Authors:** Yeong Jer Lim, Andrew D. Duckworth, Kim Clarke, Paul Kennedy, Indrani Karpha, Melanie Oates, Matthew Gornall, Nagesh Kalakonda, Joseph R. Slupsky, Andrew R. Pettitt

**Affiliations:** ^1^ Department of Molecular & Clinical Cancer Medicine, University of Liverpool, Liverpool, United Kingdom; ^2^ Haemato-oncology Department, The Clatterbridge Cancer Centre National Health Service (NHS) Foundation Trust, Liverpool, United Kingdom; ^3^ Computational Biology Facility, University of Liverpool, Liverpool, United Kingdom; ^4^ Department of Pharmacology & Therapeutics, University of Liverpool, Liverpool, United Kingdom; ^5^ Liverpool Clinical Trials Centre, University of Liverpool, Liverpool, United Kingdom

**Keywords:** chronic lymphocytic leukaemia (CLL), T cells, biomarker, chemoimmunotherapy, Tbet/TBX21 transcription factor

## Abstract

**Introduction:**

T-cell dysfunction is a hallmark of chronic lymphocytic leukemia (CLL), but the extent to which individual CD4^+^ or CD8^+^ T-cell subpopulations influence specific clinical events remains unclear. To address this knowledge gap, we utilised high-dimensional mass cytometry to profile circulating CD4^+^ and CD8^+^ T-cells in pre-treatment samples from a well-defined cohort of CLL patients undergoing initial therapy as part of a clinical trial.

**Methods:**

Pre-treatment blood samples from 138 CLL patients receiving initial chemoimmunotherapy containing bendamustine or chlorambucil in the NCRI RIAltO trial (NCT01678430; EudraCT 2011-000919-22) were subjected to deep immunophenotyping by mass cytometry using a bespoke panel of 37 antibodies. T-cell clusters were identified through unsupervised clustering and related to treatment outcomes. Additionally, a randomly selected cohort of 30 CLL patients underwent T-cell stimulation with anti-CD3/CD28 microbeads, followed by cytokine analysis using a separate 36-antibody panel, which included seven cytokines.

**Results:**

Seventeen CD4^+^ and 22 CD8^+^ T-cell clusters were identified in a discovery cohort of 79 patients. Three of these clusters, measured as a proportion of their parental CD4^+^ or CD8^+^ populations, correlated with a reduced risk of grade ≥3 infection, grade ≥3 second primary malignancy (SPM) and death, respectively. Three corresponding T-cell subpopulations prospectively defined by non-redundant markers and Boolean gating (ICOS^+^HLA-DR^+^PD1^+^TIGIT^+^Tbet^+^CD4^+^ T-helper cells; CD27^+^CD28-PD1^+^Tbet^+^Eomes^+^CD8^+^ cells; and CD27^+^CD28-GrymB^+^Tbet^+^Eomes^+^CD8^+^ terminal effector cells) showed the same clinical correlations as the clusters on which they were based. With the exception of SPM for which there were insufficient events, these correlations were confirmed in a separate validation cohort of 59 patients. *In-vitro* stimulation of a subset of CLL patients in the discovery cohort showed an enrichment of primed and polyfunctional cells in all three Tbet^+^ T-cell subpopulations of interest.

**Conclusion:**

Our study provides new insights into the potential for Tbet+ T-cell subpopulations to influence and predict specific clinical events in CLL. This, in turn, raises the possibility that these respective subpopulations could play an important role in controlling infection, solid tumours and CLL itself.

**Clinical Trial Registration:**

https://www.clinicaltrials.gov/, identifier NCT01678430; https://www.isrctn.com/ISRCTN09988575, identifier EudraCT 2011-000919-22

## Introduction

Chronic lymphocytic leukaemia (CLL) is characterised by the clonal expansion of CD5^+^ B lymphocytes, which accumulate in the blood, bone marrow, and secondary lymphoid organs. At these sites of tissue involvement, CLL cells engage in a dynamic interplay that shapes the immune microenvironment and perturbs normal immune function while simultaneously relying on non-malignant immune cells for survival and proliferation signals. In keeping with this, multiple abnormalities of circulating T-cells have been described in CLL, including a reduced CD4:CD8 ratio (1), increased regulatory T-cells (2, 3), increased differentiation towards antigen-experienced and terminally differentiated memory cells (4, 5), and increased expression of activation and exhaustion (5–7) markers.

Despite these observations, functional studies investigating the role of such T-cell abnormalities in driving CLL progression and pathogenesis have produced conflicting results (8, 9), placing considerable importance on elucidating clinical correlations. Moreover, although T-cells provide protection against both tumours and pathogens, their potential as biomarkers to predict the risk of these adverse events remains unclear. In this respect, although unsupervised, machine learning-based approaches offer significant promise for biomarker discovery among T-cells, their clinical translation is often hindered by the stochastic nature of individual results and the limited availability of omics techniques for routine clinical applications (10). Conversely, supervised analyses of broad T-cell populations in CLL provide direct clinical applicability but have yielded less clinically useful correlations (6, 11, 12), likely due to the substantial heterogeneity that exists within these populations.

In the present study, we utilised high-dimensional mass cytometry to profile circulating CD4^+^ and CD8^+^ T-cells in pre-treatment samples from a well-defined cohort of CLL patients undergoing initial therapy as part of a clinical trial. Our approach leveraged the strengths of both unsupervised and supervised methodologies by first identifying clinically significant T-cell subpopulations using an unsupervised discovery tool, and then translating these findings into a cell subpopulation that could be prospectively identified through a supervised approach. Using this methodology, we identified three Tbet^+^ T-cell subpopulations, where higher cell frequencies were associated with a reduced risk of infections, second primary malignancies (SPMs), and death. Our findings highlight the potential of specific T-cell subpopulations as pre-treatment biomarkers for CLL patients undergoing anti-CLL therapy.

## Materials and methods

### Patient selection

All patients in this study were enrolled on the NCRI phase III RIAltO trial (Randomised Investigation of Alternative Ofatumumab-based regimens for less fit patients with CLL; NCT01678430; EudraCT 2011-000919-22; Research Ethics Committee (REC) ref. 11/NW/0548). RIAltO is an open-labelled, multicentre randomised controlled trial (RCT) investigating the second generation anti-CD20 monoclonal antibody ofatumumab combined with either chlorambucil or bendamustine in patients with CLL for whom FCR was considered unsuitable (13). 521 patients were enrolled between December 2011 and April 2018 including 145 between September 2014 and March 2016 who received idelalisib (first-in-class phosphoinositide 3-kinase delta (PI3Kδ) inhibitor) or placebo in addition to chemoimmunotherapy. Follow-up data was available to April 2021. The CONSORT diagram in **Supplementary Figure S1** shows how samples were selected. The discovery cohort comprised patients who were randomised to receive bendamustine or chlorambucil, with or without idelalisib, and for whom suitable samples were available (n=79). The validation cohort comprised randomly selected patients who received bendamustine and ofatumumab only and for whom suitable samples were available (n=59). The study was approved by the UK CLL Biobank (REC ref. 19/NW/0573) and performed in accordance with the Declaration of Helsinki and relevant ethical guidelines for research in humans, with written consent obtained from all patients.

### Clinical data

Grade 3-5 infections (which included febrile neutropenia) and SPMs were defined in accordance with the Common Terminology Criteria for Adverse Events (CTCAE) v4.0. Grade 3-5 infections were reported from the point of informed consent until 6 months after the last dose of anti-CLL treatment and censored at that point in the analysis. In contrast, grade 3-5 SPM were reported until the end of the study. Short-term therapy effectiveness was measured as end of treatment (EoT) measurable residual disease (MRD) in the bone marrow quantified by flow cytometry and using cut-offs corresponding to <1 CLL cell per 10^2^ leukocytes (MRD2), <10^-3^ CLL cells (MRD3) and <10^-4^ CLL cells (MRD4) (14). Long-term therapy effectiveness was measured as time to progression (TTP) using iwCLL criteria (15).

### Sample preparation

Blood samples from CLL patients were collected in EDTA and transported within 24 hours to the UK CLL Biobank (REC ref 14/NW/1014), where peripheral blood mononuclear cells (PBMC) were isolated by centrifugation over Lymphoprep and cryopreserved in 10% DMSO at -80°C. Healthy control samples, obtained from the Liverpool Blood Disease Biobank (REC ref 16/NW/0810), were also included to enable batch alignment. Prior to analysis, samples were thawed at 37°C, diluted incrementally in ice-cold cell culture media consisting of RPMI-1640 and 10% FBS and washed before counting.

### CD19^+^ B cell depletion

PBMC were washed with purification buffer consisting of phosphate-buffered saline (PBS; pH of 7.2) supplemented with 0.5% bovine serum albumin and 2mM EDTA, resuspended with a Fc receptor blocker FcX (BioLegend, UK) at a ratio of 1:4, and incubated for 10 mins at 4°C. Anti-CD19 microbeads (Miltenyi Biotech, UK) were added and samples incubated for 15 mins at 4°C before being washed in PBS and passed through a negative selection column using the QuadroMACS™ Separator system (Miltenyi Biotech, UK).

### *Ex-vivo* T cell stimulation

Cell suspensions were washed and resuspended in cell culture medium (RPMI-1640 supplemented with Pen/Strep, L-glut, 10% FBS) at a density of 1 x 10^6^/ml and aliquoted into a 24-well plate. CD3/CD28 Dynabeads^®^ (Gibco, UK) were added at a ratio of 1:1 and the samples incubated for 48 hours at 37°C in humidified CO_2_ (0.2%). GolgiPlug™ (BD biosciences, UK) was then added to each well at a concentration of 1μl/ml and incubated for 4-6 hours at 37°C prior to analysis.

### Barcoding

Individual PBMC samples were incubated for 30 minutes at 4°C with a CD45 antibody conjugated with different combinations of ^89^Yb, ^106^Cd, ^110^Cd, ^111^Cd, ^113^Cd, ^114^Cd, ^115^In, and ^116^Cd (HI30, Biolegend). Barcoded samples were then washed (550xg/4°C/5 minutes) twice with Maxpar Cell Staining Buffer (CSB; Standard Biotools™, US) and pooled together to create a single, multiplexed sample.

### Mass cytometry (CyTOF)

Pooled samples were incubated for 45 minutes at 4°C with a cocktail of antibodies reactive to surface epitopes (**Supplementary Tables S1**, **S2**) diluted to their respective optimal concentrations, washed with CSB followed by Maxpar phosphate buffered saline (PBS; Standard Biotools™, US), resuspended in a PBS-cisplatin solution (prepared by diluting Cell-ID™ cisplatin-195Pt (Standard Biotools™, US) in PBS at a ratio of 1:1000), and incubated for 5 minutes at room temperature (RT). Cisplatin was then quenched by adding RPMI supplemented by 20% FBS at 5 times the total PBS-cisplatin volume used. Cells were then fixed by incubating overnight at 4°C with 1.6% formaldehyde (ThermoFisher Scientific, UK), permeabilised using the eBioscience™ Foxp3/Transcription Factor Staining Buffer Set (ThermoFisher Scientific, UK) as per manufacturer’s instructions, and incubated for 30 minutes at RT with a cocktail of antibodies reactive to internal epitopes (**Supplementary Tables S1**, **S2**). After washing, cells were incubated for a further 60 minutes at RT in cell intercalation solution consisting of 125nM Maxpar^®^ Intercalator-Ir and Maxpar^®^ Fix and Perm Buffer (Standard Biotools™, US) at a 1:2000 ratio. Cells were then washed with Maxpar^®^ Cell Acquisition Solution (Standard Biotools™, US) before adding EQ calibration beads a 1:10 ratio. Finally, samples were loaded into the Helios mass cytometer (Standard Biotools™, US) and cell events recorded at a rate of 200-400 cells/second. A summary of the experimental steps involved for CyTOF analysis is provided in **Supplementary Figure S2**.

### Cleaning and analysis CyTOF data

Raw data was first normalised against EQ beads using the CyTOF Software v8.0 and analysed using the online Cytobank software (Beckman-Coulter Life Sciences, US). Doublets were removed by the gating of Intercalator-Ir and Gaussian parameters while viable cells were identified by gating 195^Pt^ negative events (**Supplementary Figure S3A**). Samples were then debarcoded in the R environment using *FlowCore* and *CATALYST* package (16). Subsequently, samples were batch-aligned by applying the *CytoNorm* package (17), using designated anchor samples comprising biological replicates of a healthy donor that were included in every batch. Unsupervised analysis involving UMAP and FlowSOM was performed using the *CATALYST* package while supervised analysis using Boolean gating was performed using the Cytobank software. Heatmaps were generated using the *ComplexHeatmap* package.

### Clinical correlations

Data visualisation and statistical analyses were performed using R software. Mann-Whitney U test was used to compare differences in T cell population frequencies. Uni- and multivariable analyses of FlowSOM cluster frequencies were performed using Cox proportional hazard regression (coxph; using *survival* R package) in censored outcomes and logistic regression in uncensored outcomes. The Kaplan-Meier method and log-rank test were used when comparing cumulative risk of infections or SPMs, as well as overall survival (OS) between patient groups. A p-value of 0.05 was used throughout to determine statistical significance.

### Data sharing statement

Datasets analysed during the current study are available from the corresponding author on reasonable request.

## Results

### T-cell subpopulations vary widely among individual CLL patients

**Figure 1:**
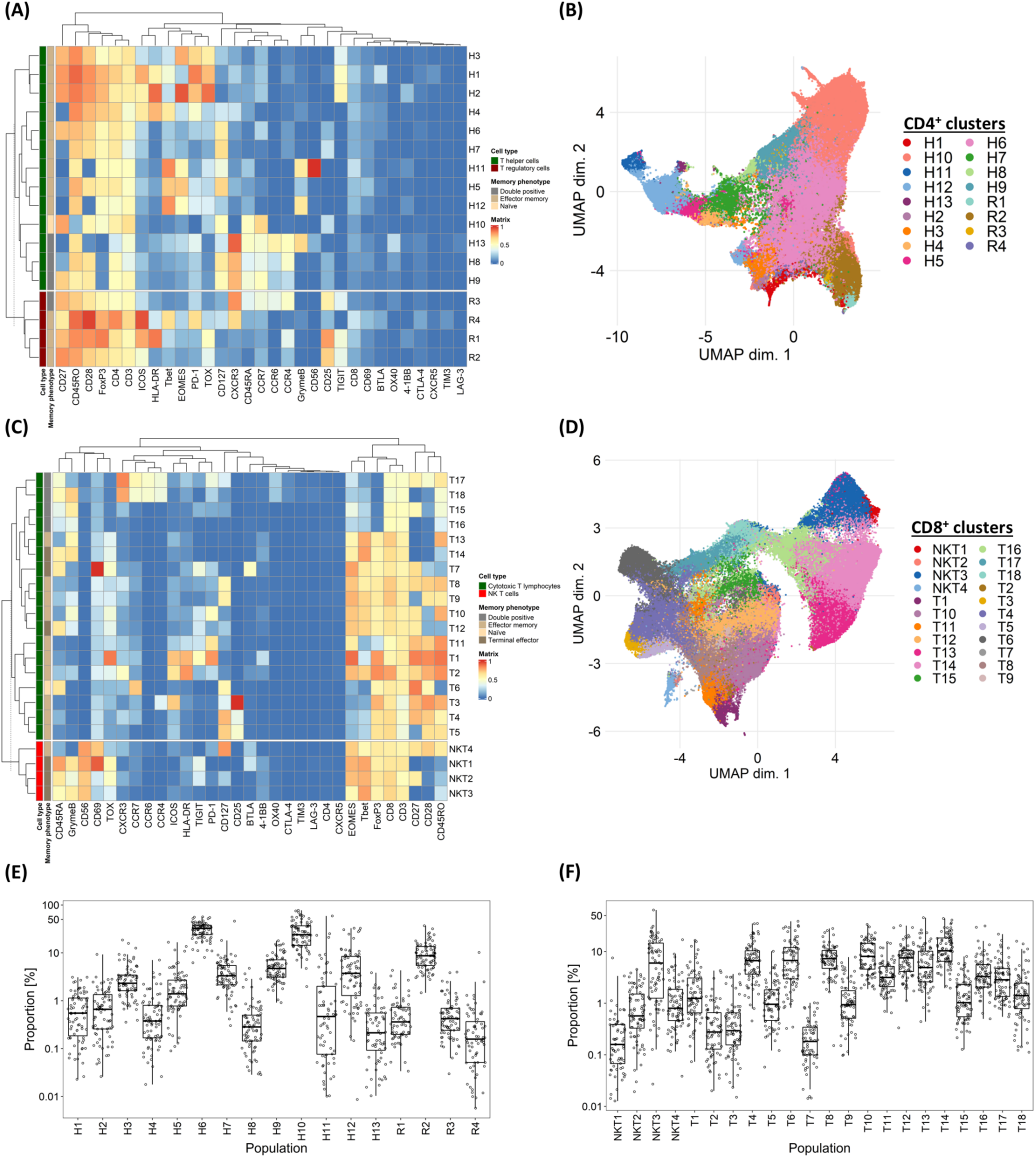
Variability in marker expressions and frequency of T-cell subpopulations among individual CLL patients. **(A)** Heatmap illustrating the median marker expression of CD4^+^ clusters identified on FlowSOM. **(B)** UMAP visualisation of CD4^+^ T-cells annotated by FlowSOM clusters. **(C)** Heatmap illustrating the median marker expression of CD8^+^ clusters identified on FlowSOM. **(D)** UMAP visualisation of CD8^+^ T-cells annotated by FlowSOM clusters. **(E)** Frequency distribution of each CD4^+^ FlowSOM cluster within the overall CD4^+^ T-cell population in each CLL patient in the discovery cohort. **(F)** Frequency distribution of each CD8^+^ FlowSOM cluster within the overall CD8^+^ T-cell population in each CLL patient in the discovery cohort. H: T helper cells, R: T regulatory cells, NKT: NK T CD8^+^ cells, T: CD8^+^ cytotoxic T-cells.

We first sought to establish the extent of variation in T-cell subpopulations among CLL patients requiring initial therapy. To do so, cryopreserved PBMCs obtained from 79 patients enrolled in the RIAltO trial (**Table 1**) were subjected to deep immunophenotyping using mass cytometry and a panel of 37 antibodies (**Supplementary Table S1**). CD4^+^ and CD8^+^ T-cells were individually organised into 30 clusters respectively using the FlowSOM clustering algorithm (18). Those clusters expressing similar markers were then merged, and minimally abundant clusters (3 or more events in fewer than 10% of samples) excluded. A detailed description of our clustering approach is provided in **Supplementary Figure S4**. Among CD4^+^ T-cells, 17 distinct clusters were identified and categorised as regulatory T-cells (Treg; R1-4) based on CD25, FoxP3 and CD127 expression, and T helper cells (Th; H1-13) for the remaining clusters. One (H10) cluster contained naïve cells (CD45RA^+^CCR7^+^), four (H8, H9, H13, R3) co-expressed CD45RO and CD45RA, while the rest comprised effector memory (EM) cells (CD45RO^+^CCR7^-^). CD8^+^ T-cells were divided into 22 clusters, categorised as NK/T-cells (NKT1-4) based on CD8 and CD56 co-expression, and cytotoxic T-cells (CTLs; T1-18) for the remaining clusters. Most clusters comprised EM cells, with the exception of one (T6) comprising naïve cells, six (NKT1-NKT3, T7, T12, T14) comprising terminal effector (TE) cells (CD45RA^+^CCR7^-^), and four (T15-T18) co-expressing CD45RO and CD45RA (**Figures 1A–D**). To understand the extent to which T-cell subpopulations varied among individual patients, the relative size of each cluster within its parental CD4^+^ or CD8^+^ population was calculated for each sample and the spread of data visualised. Significant variation was found in the size of all clusters (**Figures 1E, F**), thereby justifying correlation with clinical events.

**Table 1:** Baseline characteristics of all included patients.

Variable		Discovery cohort	Validation cohort
No. of patients		79	59
Median age [range] (years)		75 [70.5-79]	76 [60-88]
Sex	Male	62 (78.5%)	41 (69.5%)
	Female	17 (21.5%)	18 (30.5%)
Performance status	0-1	71 (89.9%)	53 (89.8%)
	2-3	8 (10.1%)	6 (10.2%)
Binet stage	A	12 (15.2%)	11 (18.6%)
	B	25 (31.6%)	17 (28.8%)
	C	42 (53.2%)	30 (50.8)
	Unknown	0 (0%)	1 (1.7%)
FISH status	TP53 deletion	1 (1.3%)	4 (6.8%)
	ATM deletion	11 (13.9%)	8 (13.6%)
	13q deletion	40 (50.6%)	30 (50.8%)
	Trisomy 12	18 (22.8%)	6 (10.2%)
	Unknown	2 (2.5%)	1 (1.7%)
IGHV status	Mutated	26 (32.9%)	24 (40.7%)
	Unmutated	34 (43.0%)	27 (45.8%)
	Unknown	19 (24.1%)	8 (13.6%)
Chemotherapy allocation	Bendamustine	41 (51.9%)	59 (100%)
	Chlorambucil	38 (48.1%)	0 (0%)
Additional idelalisib randomisation	Idelalisib	35 (44.3%)	N/A*
	Placebo	44 (55.7%)	N/A*

*Patients included in the validation cohort were enrolled before the amendment to RIAltO, which permitted additional randomisation between receiving either idelalisib or placebo.

### A distinct subpopulation of Tbet^+^ CD4^+^ T-helper cells is linked to reduced risk of grade ≥3 infections

**Figure 2:**
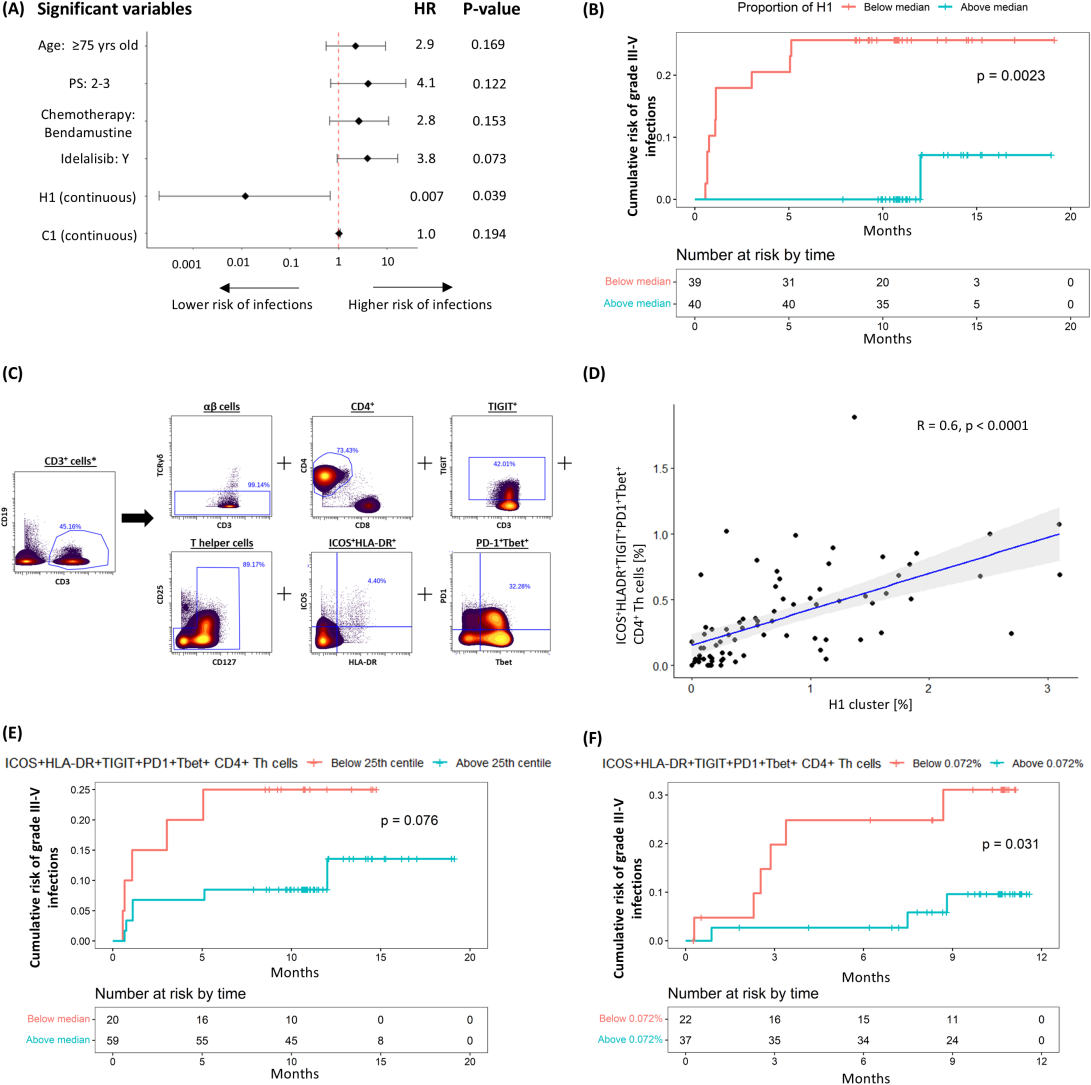
Negative correlation between H1 cluster (ICOS^+^HLA-DR^+^PD1^+^TIGIT^+^Tbet^+^CD4^+^ Th cells) and risk of subsequent grade ≥3 infection. **(A)** Multivariable coxph analysis of the association between grade 3-5 infections and baseline characteristics, treatment allocation and T-cell clusters found to be significant on univariable analysis. **(B)** Kaplan-Meier (K-M) plot of cumulative risk of infection among patients with pre-treatment H1 above (≥0.53%) or below (<0.53%) the median in the discovery cohort. **(C)** Gating strategy used to identify ICOS^+^HLA-DR^+^PD1^+^TIGIT^+^Tbet^+^ CD4^+^ Th cells. **(D)** Scatter plots and linear regression analyses of the association between H1 and ICOS^+^HLA-DR^+^PD1^+^TIGIT^+^Tbet^+^ CD4^+^ Th cells. K-M plots of cumulative risk of infection among patients with high (≥0.072%) vs low (<0.072%) ICOS^+^HLA-DR^+^PD1^+^TIGIT^+^Tbet^+^ CD4^+^ Th cells prior to treatment in the **(E)** discovery and **(F)** validation cohort. *Gated from live singlets (**Supplementary Figure S3A**).

We first explored the association between pre-treatment T-cell subpopulations and the subsequent occurrence of severe infections. Univariable coxph analysis, incorporating all relevant co-variates, identified an association between the risk of subsequent infections and pre-treatment haemoglobin levels, as well as the pre-treatment frequencies of the C1 and H1 CD4^+^ clusters. However, only the H1 cluster was considered further as it consistently retained significance in multivariable analyses, particularly after adjusting for clinically significant covariates such as age, performance status and treatment allocation (**Supplementary Table S3**, **Figure 2A**). In line with this, stratification of patients based on the median size of the H1 cluster showed that those with a higher frequency of H1 cells had a significantly lower risk of grade ≥3 infections (HR 0.082 [95% CI: 0.01 – 0.643], P = 0.002; **Figure 2B**). Given the heterogeneity of the analysed CLL cohort, which included patients with different baseline characteristics receiving different treatment regimens, we performed a sensitivity analysis confirming that the pre-treatment H1 cluster size, using the median cut-off point, independently predicted infection risks despite adjusting for baseline demographics, CLL disease characteristics, treatment allocation and subsequent disease response (**Supplementary Table S4**).

When examining the marker expression profile of H1, we found that it consisted of non-senescent (CD27^+^CD28^+^) effector memory (EM; CD45RO^+^CCR7^-^) Th cells co-expressing activation markers ICOS and HLA-DR, exhaustion markers PD-1, TIGIT and TOX, and the T-box transcription factor T-bet. To define this subpopulation prospectively, we employed a Boolean gating strategy that reflected the cluster’s main non-redundant features (ICOS^+^HLA-DR^+^PD1^+^TIGIT^+^Tbet^+^ Th; **Figure 2C**). A significant correlation was observed between the size of the H1 cluster and that of the prospectively defined subpopulation (R^2^ = 0.6, P < 0.0001; **Figure 2D**), thereby validating the gating strategy. In keeping with our findings for H1, there was a trend for a lower risk of grade ≥3 infection in patients with a greater proportion (25^th^ centile or higher; ≥ 0.072%) of ICOS^+^HLA-DR^+^PD1^+^TIGIT^+^Tbet^+^ Th cells (HR 0.358 [95% CI: 0.109 – 1.186], P = 0.076; **Figure 2E**). These findings were further validated in a separate cohort of 59 patients (**Table 1**, validation cohort) using the same methodology, gating strategy and cut-off value, confirming an association between ICOS^+^HLA-DR^+^PD1^+^TIGIT^+^Tbet^+^ Th cells and a lower risk of grade ≥3 infections (HR 0.245 [95% CI: 0.061 – 0.981], P = 0.031; **Figure 2F**).

### A distinct subpopulation of Tbet^+^ CD8^+^ effector memory cells is linked to reduced risk of grade ≥3 SPMs

**Figure 3:**
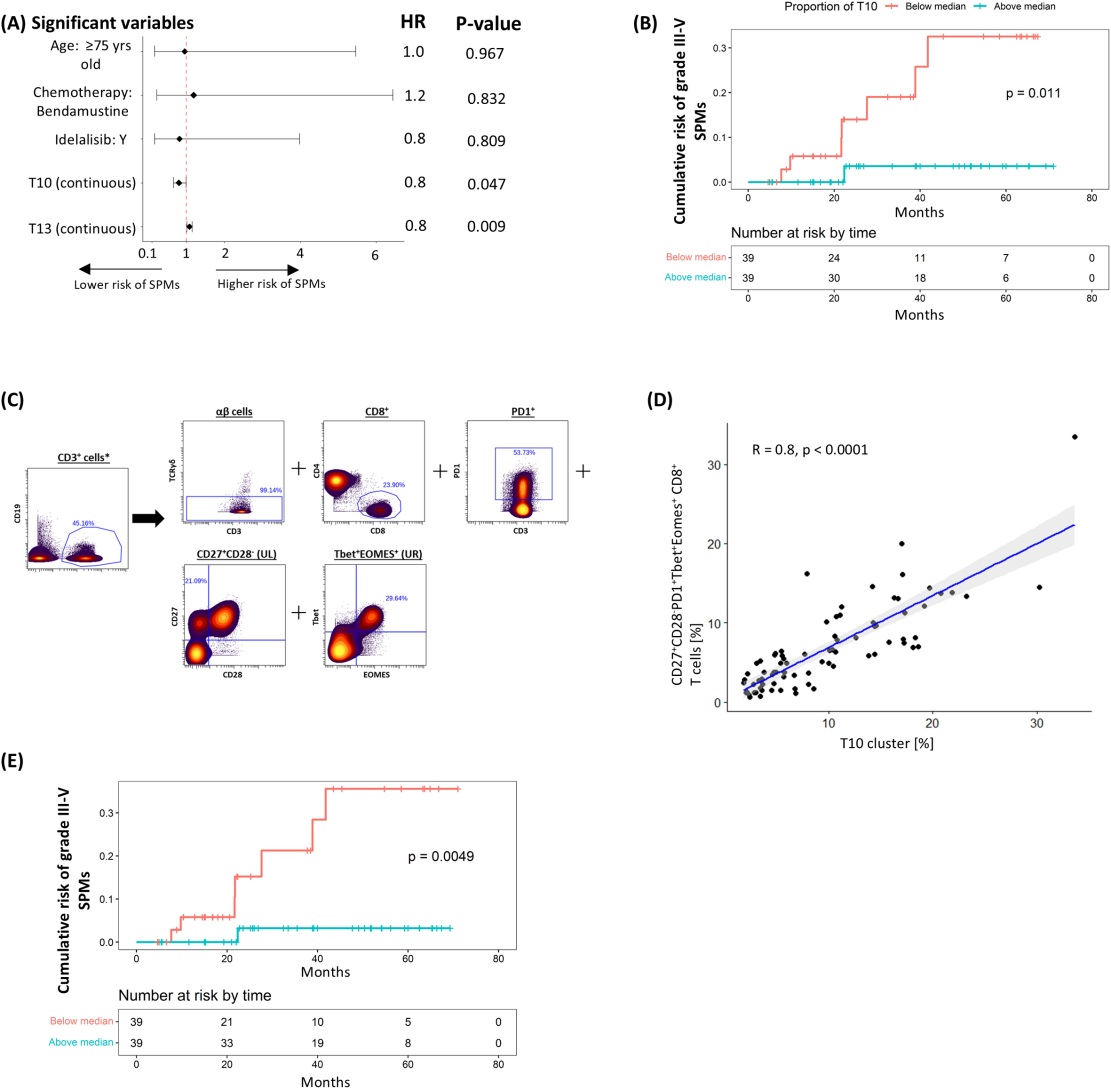
Negative correlation between T10 cluster (CD27^+^CD28^-^PD1^+^Tbet^+^Eomes^+^ CD8^+^ T-cells) and risk of subsequent grade ≥3 SPM. **(A)** Multivariable coxph analysis of the association between grade 3-5 SPMs and baseline characteristics, treatment allocation and T-cell clusters found to be significant on univariable analysis. **(B)** K-M plot of cumulative risk of SPM among patients with pre-treatment T10 above (≥8.1%) or below (<8.1%) the median in the discovery cohort. **(C)** Gating strategy used to identify CD27^+^CD28^-^PD1^+^Tbet^+^Eomes^+^ CD8^+^ T-cells. **(D)** Scatter plots and linear regression analyses of the association between T10 and CD27^+^CD28^-^PD1^+^Tbet^+^Eomes^+^ CD8^+^ T-cells. **(E)** K-M plot of cumulative risk of infection among patients with high (≥5.8%) vs low (<5.8%) CD27^+^CD28^-^PD1^+^Tbet^+^Eomes^+^ CD8^+^ T-cells prior to treatment in the discovery cohort. *Gated from live singlets (**Supplementary Figure S3A**).

We next explored the association between pre-treatment T-cell subpopulations and the subsequent occurrence of grade ≥3 SPMs using the same analytical approach. Univariable analysis showed that pre-treatment frequencies of clusters R2, T10, and T13 correlated with the risk of SPMs, though only T10 consistently retained significance in the multivariable analyses (**Supplementary Table S5**, **Figure 3A**). Stratifying patients based on the median cluster size showed that those with higher T10 levels had a significantly lower risk of grade ≥3 SPMs (HR 0.108 [95% CI: 0.013 – 0.881], P = 0.011; **Figure 3B**). Sensitivity analysis confirmed that the pre-treatment T10 cluster size, using the median cut-off point, independently predicted risk of SPMs despite adjustments for baseline demographics, CLL disease characteristics, treatment allocation and subsequent disease response (**Supplementary Table S6**).

Examination of the marker expression profile of T10 revealed that it comprised EM (CD45RO^+^CCR7^-^) CTLs co-expressing exhaustion markers PD-1, TIGIT and TOX, cytotoxic marker Granzyme B, and T-box transcription factors T-bet and Eomes. The loss of CD28 also suggests a shift towards a senescent phenotype, however further confirmation with CD57 and KLRG-1 is required. When a Boolean gating strategy reflecting its main non-redundant features (CD27^+^CD28^-^PD1^+^Tbet^+^Eomes^+^ CD8^+^; **Figure 3C**) was applied, a significant correlation was observed with the size of the T10 cluster (R^2^ = 0.82, P < 0.0001; **Figure 3D**). In line with our findings for T10, patients with more (50^th^ centile or higher; ≥ 5.8%) CD27^+^CD28^-^PD1^+^Tbet^+^Eomes^+^ CD8^+^ cells exhibited a significantly lower risk of developing grade ≥3 SPM (HR 0.089 [95% CI: 0.011 – 0.728], P = 0.005; **Figure 3E**). However, validation of these results was not possible due to the limited number of grade ≥3 SPMs in the validation cohort, where only two patients were affected.

### A distinct subpopulation of Tbet^+^ CD8^+^ terminal effector cells is linked to improved overall survival

**Figure 4:**
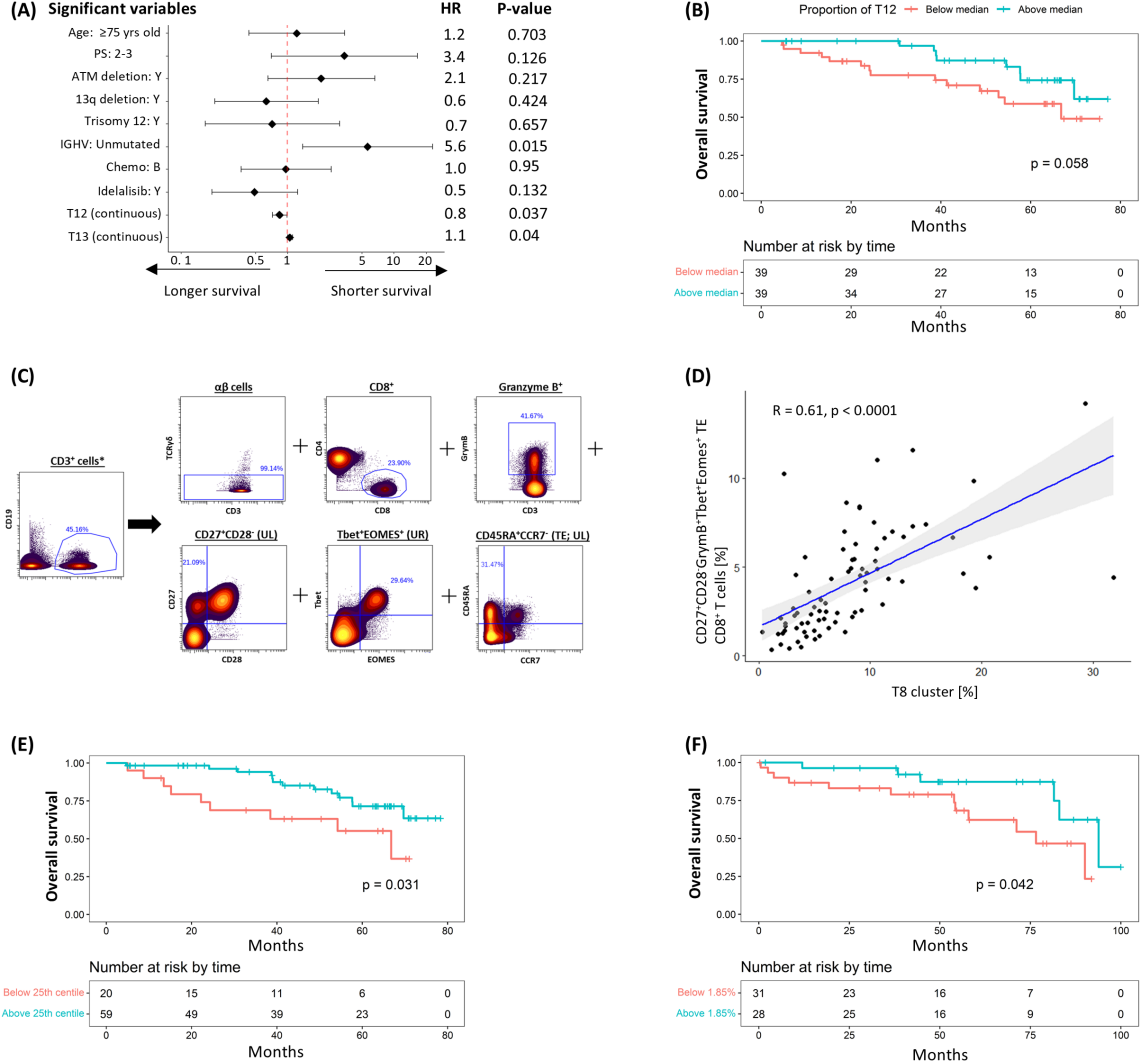
Negative correlation between T12 cluster (CD27^+^CD28^-^GrymB^+^Tbet^+^Eomes^+^ TE CD8^+^ T-cells) and risk of subsequent death. **(A)** Multivariable coxph analysis of the association between overall survival (OS) and baseline characteristics, CLL prognostic factors, treatment allocation and T-cell clusters found to be significant on univariable analysis. **(B)** K-M plot of cumulative risk of death among patients with pre-treatment T12 above (≥7.6%) or below (<7.6%) the median in the discovery cohort. **(C)** Gating strategy used to identify CD27^+^CD28^-^GrymB^+^Tbet^+^Eomes^+^ terminal effector (TE) CD8^+^ T-cells. **(D)** Scatter plots and linear regression analyses of the association between T12 and CD27^+^CD28^-^GrymB^+^Tbet^+^Eomes^+^ TE CD8^+^ T-cells at pre-treatment. K-M plots of cumulative risk of death among patients with high (≥1.85%) vs low (<1.85%) CD27^+^CD28^-^GrymB^+^Tbet^+^Eomes^+^ TE CD8^+^ T-cells in the **(E)** discovery and **(F)** validation cohort. *Gated from live singlets (**Supplementary Figure S3A**).

The relationship between pre-treatment T-cell populations and overall survival was subsequently examined. In the univariable analysis, IGHV mutational status, haemoglobin levels and pre-treatment cluster sizes of T12 and T13 showed borderline to significant correlations with OS; however, only IGHV and T12 consistently retained statistical significance in the multivariable analyses (**Figure 4A**; **Supplementary Table S7**). In keeping with this, stratification of patients into two groups based on the median cluster size indicated a trend towards longer survival in the group with higher T12 levels (HR: 0.440 [95% CI: 0.184 – 1.051], P = 0.058; **Figure 4B**). This trend persisted despite adjustments for baseline demographics, CLL disease characteristics, treatment allocation and subsequent disease response in the sensitivity analysis (**Supplementary Table S8**).

The T12 cluster consisted of TE (CD45RA^+^CCR7^-^) TOX^lo^GrymB^+^Tbet^+^Eomes^+^ CTLs exhibiting features (CD27^+^CD28^-^) suggestive of cell senescence. To prospectively define this T-cell subpopulation, a Boolean gating strategy was applied (CD27^+^CD28^-^GrymB^+^Tbet^+^Eomes^+^ CD8^+^ TE; **Figure 4C**), where a significant correlation between the size of this prospectively defined subpopulation and that of T12 was observed (R^2^ = 0.61, P = <0.0001; **Figure 4D**). Consistent with our previous findings, separation of patients based on the 25^th^ centile value (1.85%) showed that survival was significantly longer in the group with more CD27^+^CD28^-^GrymB^+^Tbet^+^Eomes^+^ CD8^+^ TE T-cells (HR: 0.404 [95% CI: 0.173 – 0.947], P = 0.031; **Figure 4E**). A similar correlation was also observed when the same gating strategy/cut-off value was applied to pre-treatment samples from the validation cohort (HR: 0.352 [95% CI: 0.123 – 1.005], P = 0.042; **Figure 4F**), confirming the association between this subpopulation and OS.

### T-cell subpopulations are not associated with therapy effectiveness

We next sought to elucidate any correlations between T-cell subpopulations and the effectiveness of the CLL treatment administered. Each cluster was related to EoT MRD status as a measure of initial cytoreduction, and time to progression as a measure of sustained effectiveness (**Supplementary Table S9**). Three clusters were identified that separately correlated with the attainment of MRD2 (H6), MRD3 (NKT3) and MRD4 (T1), respectively. However, since none of these clusters correlated with the attainment of MRD at more than one level, they were not considered clinically significant. Similarly, no correlation was seen between any cluster and TTP (**Supplementary Table S10**).

### Clinically significant T-cell subpopulations are enriched with primed, polyfunctional T-cells

**Figure 5:**
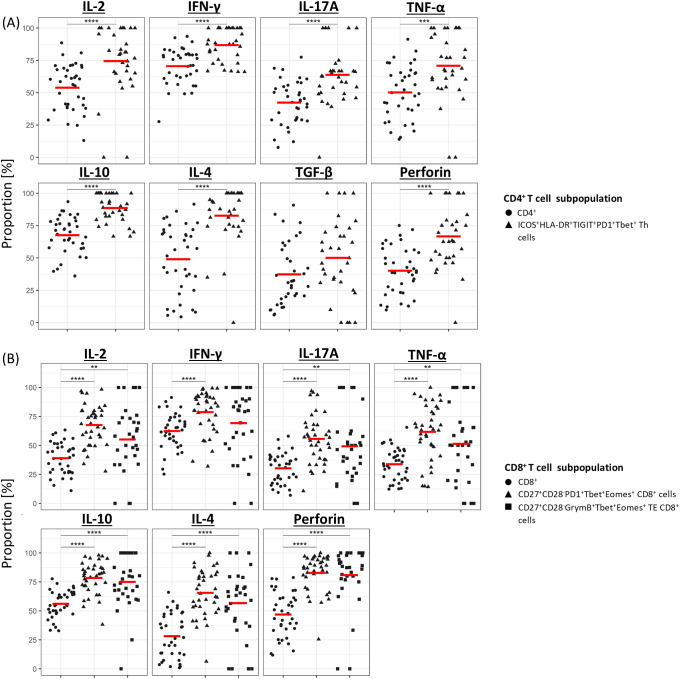
Cytokine profile of clinically significant T-cell subpopulations. Median proportion of cytokine-expressing CD4^+^ **(A)** and CD8^+^ **(B)** T cells following stimulation with anti-CD3/CD28 microbeads. For each cytokine, results are compared between the overall CD4^+^ or CD8^+^ population and the subpopulation(s) of interest. Gating strategy used to identify cytokine-expressing cells is shown in **Supplementary Figure S3B**. **P<0.01, ***P<0.001, ****P<0.0001.

To gain insight into the functional characteristics of the three clinically significant T-cell subpopulations identified in this study, PBMCs from a randomly selected subset of 30 CLL patients in the discovery cohort were stimulated using anti-CD3/CD28 microbeads and analysed for the expression of seven different cytokines using a modified CyTOF antibody panel (**Supplementary Table S2**). Apart from TGF-β in ICOS^+^HLA-DR^+^PD1^+^TIGIT^+^Tbet^+^CD4^+^ Th cells and IFN-γ in CD27^+^CD28^-^GrymB^+^Tbet^+^Eomes^+^ TE CD8^+^ T cells, the expression of pro- and anti-inflammatory cytokines was significantly higher in all three clinically significant T-cell subpopulations compared to their parental CD4^+^ or CD8^+^ populations (**Figure 5**).

**Figure 6:**
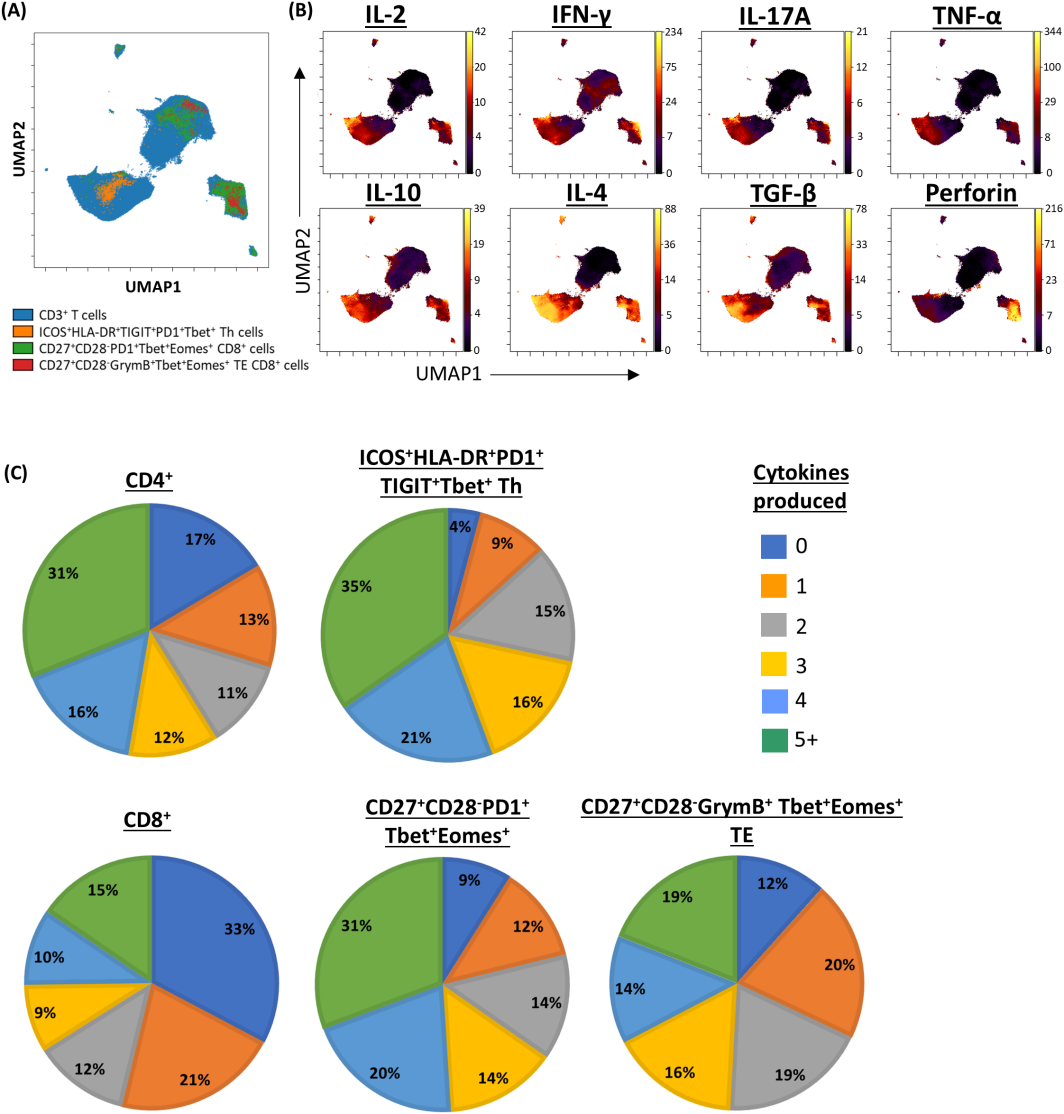
Pooled analysis of multiple cytokine expression in clinically significant T-cell subpopulations. **(A)** UMAP representing CD3/CD28-stimulated CD3^+^ T-cells from patients with CLL, annotated by clinically significant T-cell subpopulations. **(B)** UMAPs showing the expression of cytokines of interest. **(C)** Pie charts showing the proportion of cell secreting between zero and five or more cytokines within clinically significant subpopulations, compared to their respective parent populations.

**Figure 7:**
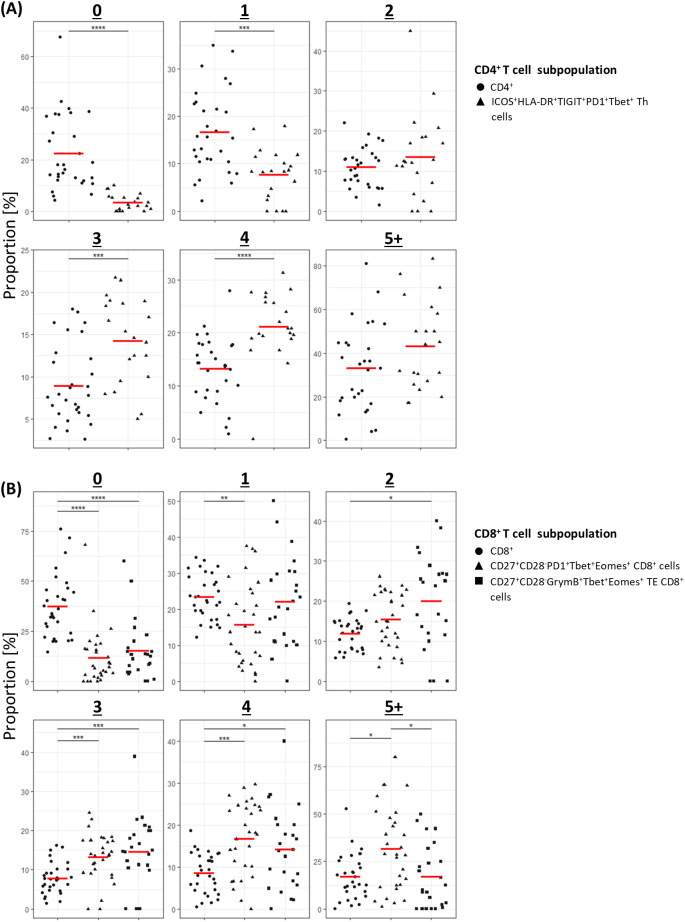
Individual patient analysis of multiple cytokine expression in clinically significant T-cells populations. **(A)** CD4^+^ T-cells; **(B)** CD8^+^ T-cells. For each number of simultaneously expressed cytokines, results are compared between the parental CD4^+^ or CD8^+^ population and the subpopulation(s) of interest. *P<0.05, **P<0.01, ***P<0.001, ****P<0.0001.

Given the role of polyfunctional T-cells in eradicating pathogen and tumour (19–22), we next sought to establish the proportion of cells in the clinically significant T-cell subpopulations that expressed multiple cytokines. Pooled analysis of all CLL samples by UMAP showed that all three subpopulations were enriched with cells co-expressing two or more cytokines (**Figure 6**; **Supplementary Figure S5**). Furthermore, analysis of cytokine expression in individual samples showed that a significantly higher proportion of cells within the clinically significant Th subpopulation expressed three or more cytokines compared to the parent CD4^+^ population, and that cells co-expressing two or more cytokines were over-represented within the two clinically significant CTL subpopulations compared to the parent CD8^+^ population. Moreover, the CTL subpopulation associated with a lower risk of SPM showed enrichment of cells co-expressing four or more cytokines (**Figure 7**). When focussing solely on pro-inflammatory cytokines (IFN-γ, TNF-α, and IL-2), clinically significant T-cell subpopulations exhibited a lower proportion of cells lacking any pro-inflammatory cytokine expression, along with a trend towards a higher proportion of cells co-expressing two or more pro-inflammatory cytokines, compared with their parental CD4 or CD8 populations (**Supplementary Figure S6**). Together, these findings indicate that all three clinically significant T-cell subpopulations are enriched with cells that are primed and, upon stimulation, exhibit poly-functional properties.

## Discussion

This study sought to explore potential connections between pre-treatment T-cell subpopulations and subsequent clinical events in patients with CLL commencing initial therapy. Our approach exploited the advent of high-dimensional mass cytometry for deep immunophenotyping, coupled with the availability of high-quality samples and associated clinical data obtained as part of a large clinical trial. Three distinctive T-cell subpopulations were identified that were associated with a reduced risk of grade ≥3 infection, grade ≥3 SPM, or death, respectively. With the exception of SPM for which there were insufficient events, these clinical correlations were confirmed in a separate validation cohort. All three T-cell subpopulations expressed both pro- and anti-inflammatory cytokines at higher levels than their respective CD4^+^ or CD8^+^ parent populations and included more cells that expressed multiple cytokines.

The use of samples obtained from the RIAltO trial ensured that all patients included in the study met the formal inclusion and exclusion criteria and were at the same point in the course of their disease as defined by the need for initial therapy, thereby minimising confounding variables resulting from disease status, prior treatments, and factors unrelated to CLL. It also ensured that the clinical data with which the samples were annotated was reliable and complete, and that the samples themselves were collected, transported, processed and stored in accordance with Good Clinical Practice, thereby optimising the quality and reliability of the laboratory data.

Our analytical approach capitalised on the strengths of mass cytometry in multidimensional single-cell analysis, while also ensuring that the findings generated remain translatable for potential clinical application. The unsupervised, simultaneous assessment of 37 different markers at a single-cell level facilitated the identification of highly specific T-cell subpopulations defined not only by their T-cell subset but also by memory, exhaustion, senescence and activation status, as well as the expression of functionally important transcription factors – something that is unlikely to be achieved through an exclusively supervised approach. On the other hand, the supervised translation of clinically significant data-dependent clusters into prospectively identifiable cell subpopulations not only offers internal validation of the clustering algorithm employed, but more importantly, enables further external validation of their significance in a separate patient cohort, as well as T-cell stimulation studies to assess their functional states, all of which supported the role of the identified T-cell subpopulations as independent predictors of infection, SPMs and death.

In keeping with a role in immune surveillance, it is notable that all three clinically significant subpopulations expressed increased levels of multiple cytokines and were enriched for cells that displayed polyfunctional properties following *in-vitro* stimulation. This primed, polyfunctional phenotype could be explained by the fact that all three subpopulations expressed Tbet, a transcription factor encoded by the *TBX21* gene, which is known to accumulate in CLL (23), and is associated with increased production of the pro-inflammatory cytokine IFNγ (24–27), increased expression of the IL-2 receptor (26), and protection against viral and bacterial infections in multiple murine models (24, 28, 29).

The CD4^+^ Th cell subpopulation associated with a reduced risk of grade ≥3 infection also expressed activation markers ICOS and HLA-DR, supporting the idea that these cells were primed and activated (30–32) for a Th1 response. This idea is in keeping with numerous studies implicating ICOS as a context- and microbe-dependent modulator of Th1 and Th2 responses (33–35). Given these considerations, the fact that these cells also expressed PD-1 and TIGIT likely reflected their state of activation rather than cellular exhaustion.

Another finding of significance is that the two clinically significant CD8^+^ T-cell subpopulations both expressed a second Tbox transcription factor, Eomes (or Tbr2), in addition to Tbet. Notably, co-expression of T-bet and Eomes has been reported to enhance the immune surveillance activity of CD8^+^ T-cells by increasing their production of IFN-γ, their cytotoxic potential, and their ability to infiltrate into the tumour microenvironment (36–38).

The fact that both of the clinically significant CD8^+^ T-cell subpopulations displayed features of an intermediate senescence phenotype (CD27^+^CD28^-^) may also be relevant as this suggests that the cells could possess both the self-renewing potential of non-senescent (CD27^+^CD28^+^) cells allowing them to persist and expand, as well as the cytotoxic capability of highly differentiated (CD27^-^CD28^-^) cells allowing them to fulfil their effector function (39, 40).

In addition to sharing certain features, the two CD8^+^ T-cell subpopulations also differed from one another in several respects. Specifically, the subpopulation associated with a lower risk of grade ≥3 SPM had an effector memory phenotype (CD45RO^+^CCR7^-^) and expressed PD1, whereas the subpopulation associated with a lower risk of death had a terminal effector phenotype (CD45RA^+^CCR7^-^) and expressed granzyme B.

As with the clinically significant CD4^+^ subpopulation, expression of PD1 in the CTL subpopulation associated with a lower risk of SPM likely resulted from its primed/activated state. Furthermore, the role of PD-1^+^ T cells in immune surveillance is supported by recent studies showing that that tumour-reactive, neoantigen-specific CTLs expressing PD-1 are enriched among tumour-infiltrating or circulating CTLs in haematological malignancies (41, 42) and solid organ cancers (43–46).

The terminal effector phenotype of the CD8^+^ subpopulation associated with a lower risk of death is in keeping with its high expression of perforin following *in-vitro* stimulation and indicates that these cells are particularly well primed for cytotoxicity. Given that patient death in CLL usually results from the direct or indirect effects of clonal expansion, evolution or transformation, this in turn suggests that such cytotoxic functionality may exert a controlling effect on the CLL clone. The fact that this CTL subpopulation was not associated with TTP following initial therapy suggests that any such controlling effects likely come into play later during the course of the disease.

There were inevitable limitations to our study. First, the T-cell profile at the onset of adverse events is not known as samples were not collected at this timepoint; this weakens any conclusions that might be drawn regarding a causative link between T-cell subsets and adverse events. Second, the simultaneous identification of multiple markers required to define the T-cell subpopulations is currently not possible with the four-color flow cytometers currently employed in most clinical laboratories, potentially posing a barrier to biomarker development and implementation. However, as the use of multiparameter flow and mass cytometry becomes more widespread and costs decrease, this concern is likely to diminish. Third, although our study demonstrated that clinically significant Tbet^+^ T-cell subpopulations can be identified following T-cell stimulation and exhibit polyfunctional properties, it remains unclear whether these cells correspond precisely to the same subpopulations identified prior to stimulation. Additionally, the phenotypic changes that may occur within these Tbet^+^ subpopulations following stimulation are also uncertain and may have clinical implications which warrants further investigations. Lastly, since the completion of RIAltO, the therapeutic landscape of CLL has shifted significantly with the introduction of BTK and BCL-2 inhibitors, such that chemoimmunotherapy no longer have a significant role in the treatment of CLL. Nevertheless, the fact that our findings were largely unaffected by the type of chemotherapy employed or the addition of idelalisib suggests that they may be applicable in other therapeutic contexts. At the very least, our findings provide proof-of-concept that selected tightly defined T-cell subpopulations precede, correlate with, and therefore potentially influence, specific clinical events in CLL.

These speculations highlight other potential directions for future work. Although not the primary focus of our study, FoxP3/CD127 co-expression was observed in a number of non-Treg clusters, where Tregs were defined by the expression of FoxP3 or CD25 in the absence of CD127 (47–49). Additionally, stimulation increased the frequency of T cells expressing immunosuppressive cytokines, even within the clinically significant subpopulations expressing Tbet which is usually associated with Th1 function. While similar findings have been reported both within (8, 50–52) and outside (53–56) the CLL context, the significance of these observations and underlying mechanisms warrants further investigation.

Given the demonstrated association between circulating T-cell phenotypes and susceptibility to infections and tumour in other settings (57–60), our findings may also be relevant to diseases beyond CLL, noting that bendamustine-containing chemoimmunotherapy is widely used as initial treatment for indolent non-Hodgkin lymphoma. In this context, deep phenotypic analysis of circulating T-cells could serve as a tool to assess a patient’s overall susceptibility to therapy-related adverse events such as infections or secondary malignancies, which may inform surveillance strategies and preventative interventions.

In conclusion, our study provides the first clinical evidence that distinctive subpopulations of Tbet^+^ T-cells influence the risk of infection, SPM and death in the setting of initial chemoimmunotherapy for CLL. This, in turn, raises the possibility that these respective subpopulations could play an important role in controlling infection, solid tumours and CLL itself. Further studies are now warranted to confirm and expand these findings in other clinical and therapeutic contexts.

## Data Availability

The raw data supporting the conclusions of this article will be made available by the authors, without undue reservation.
