# Supplementary material for: Influence of polyfunctional Tbet+ T cells on specific clinical events in chronic lymphocytic leukaemia
Source: Front Immunol. 2025 Apr 17;16:1528405. doi: 10.3389/fimmu.2025.1528405 (PMC12043603; doi:10.3389/fimmu.2025.1528405)
Supplement: Supplementary file 1 [file Table1.docx]

**Supplementary Figures:**

(A)

(B)


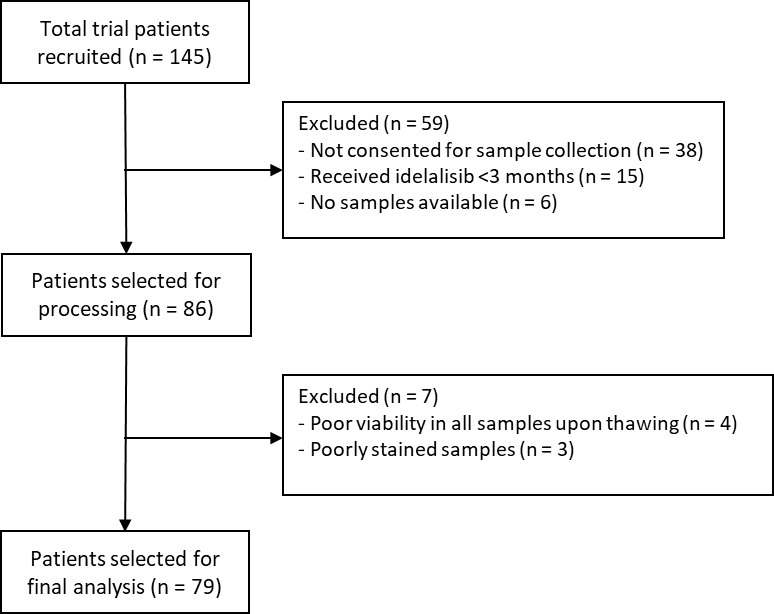

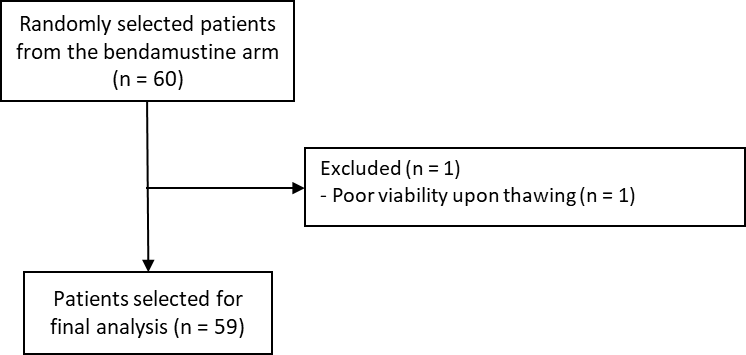


**Figure S1:** CONSORT diagram showing the inclusion of samples in the (A) discovery and (B) validation cohort.


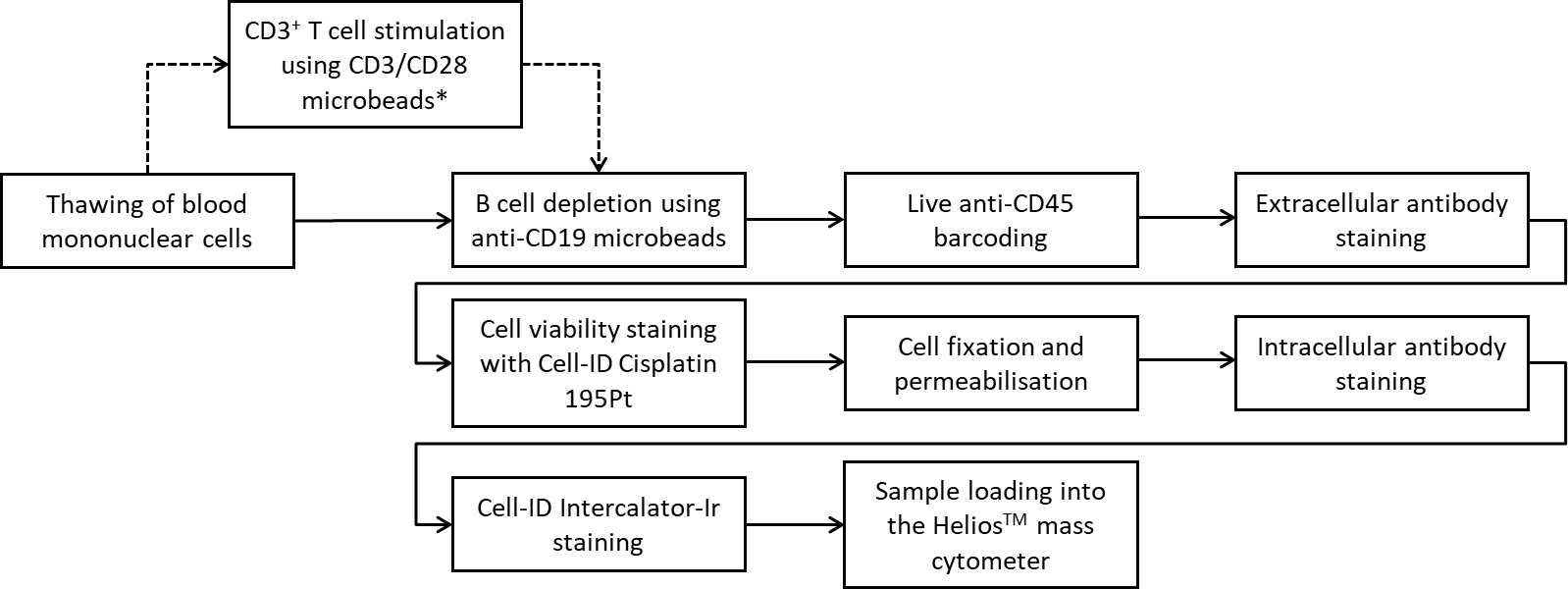


**Figure S2:** Summary of experimental steps involved in the CyTOF analysis of blood mononuclear cells of patients recruited into the RIAltO trial. *Applied to samples selected for *ex vivo* T cell stimulation.


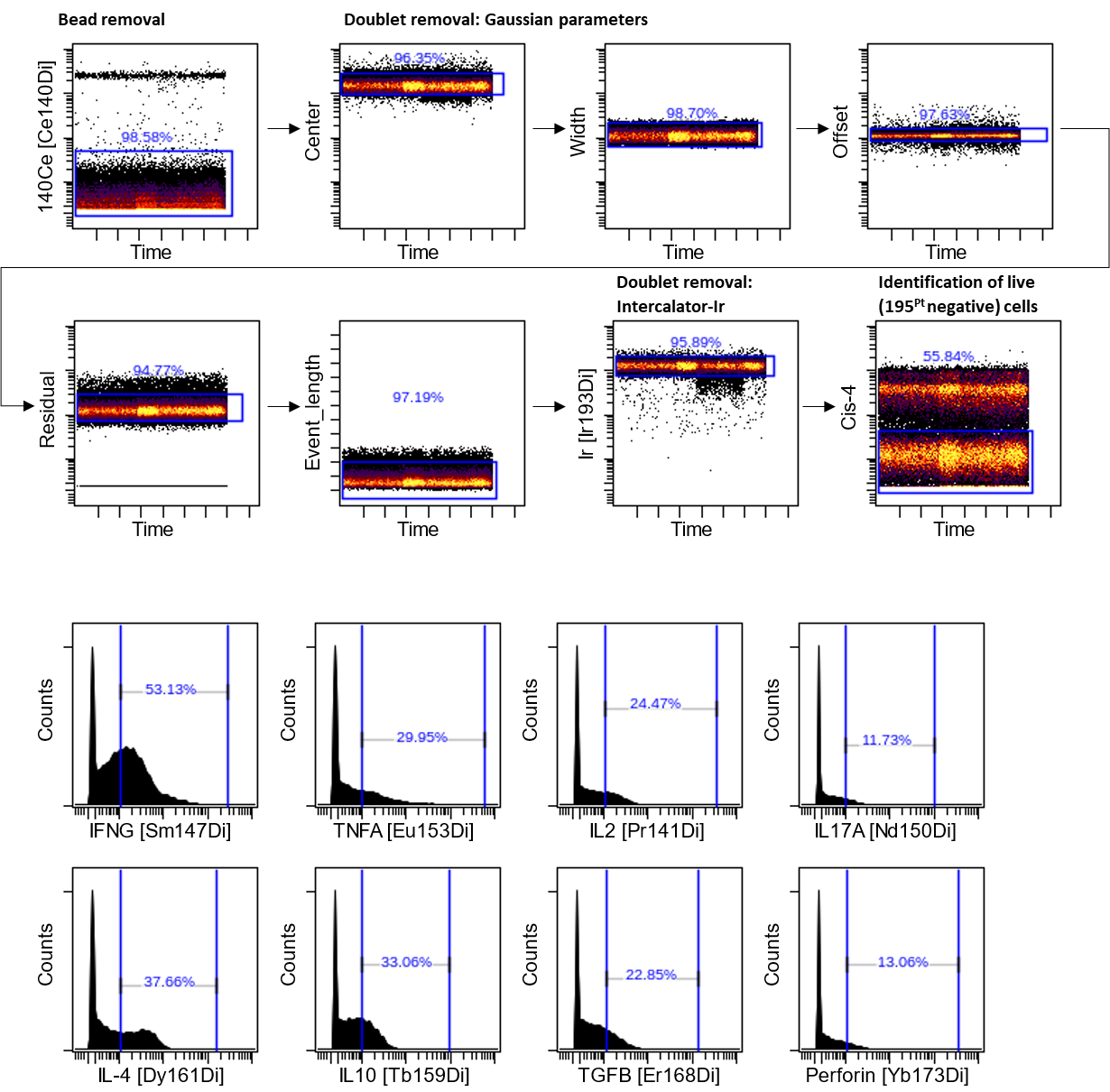


(B)

(A)

**Figure S3:** Gating strategy used to identify (A) live singlets and (B) cytokine-expressing cells.


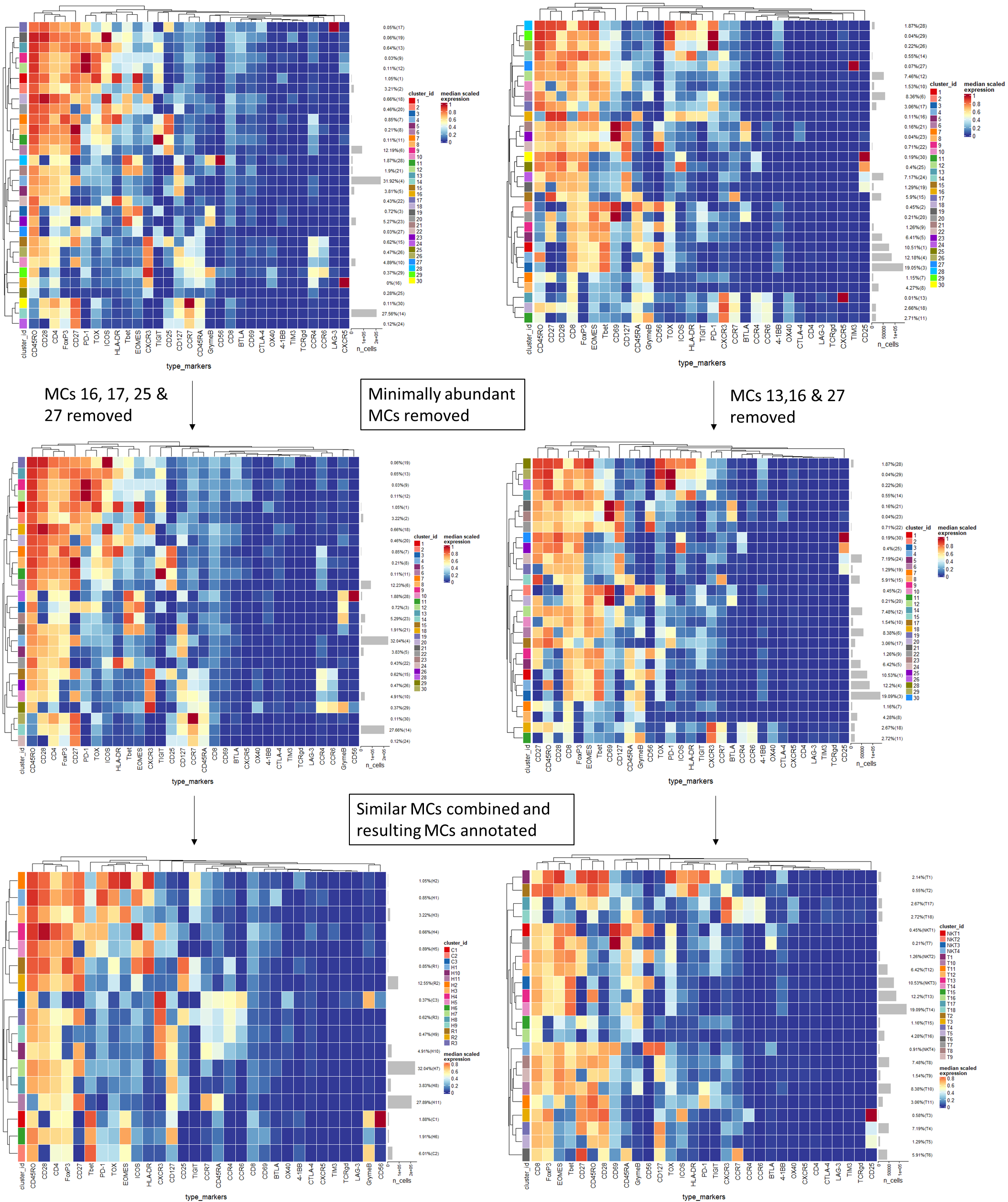


**Figure S4:** Analytical workflow of CD4^+^ (left) and CD8^+^ T cells (Right). As part of the FlowSOM algorithm, T cells were mapped into a 15x15 self-organizing map (SOM) and categorised into 225 clusters based on their proximity to nodes within the SOM. These SOM nodes will undergo additional clustering into 30 distinct clusters using hierarchical consensus clustering. Minimally abundant FlowSOM clusters – defined as those with 3 or more events in fewer than 10% of samples – were first removed and those with similar median marker expression profiles were then combined. The resultant clusters were then assigned an identification code based on their phenotype. Among CD4^+^ cells, cytotoxic T cells (“C”) were identified by the expression of Granzyme B and Tregs (“R”) by their CD25^+/-^CD127^-^FoxP3^+^ phenotype; all remaining CD4^+^ cells were classified as helper T cells (“H”). Among CD8^+^ cells, NK T cells (“NKT”) were identified by co-expression of CD8 and CD56, while all remaining cells were classified as cytotoxic T lymphocytes (“T”). In total, 0.37% CD4^+^ and 0.18% CD8^+^ events were categorized as minimally abundant clusters and excluded from the analysis.


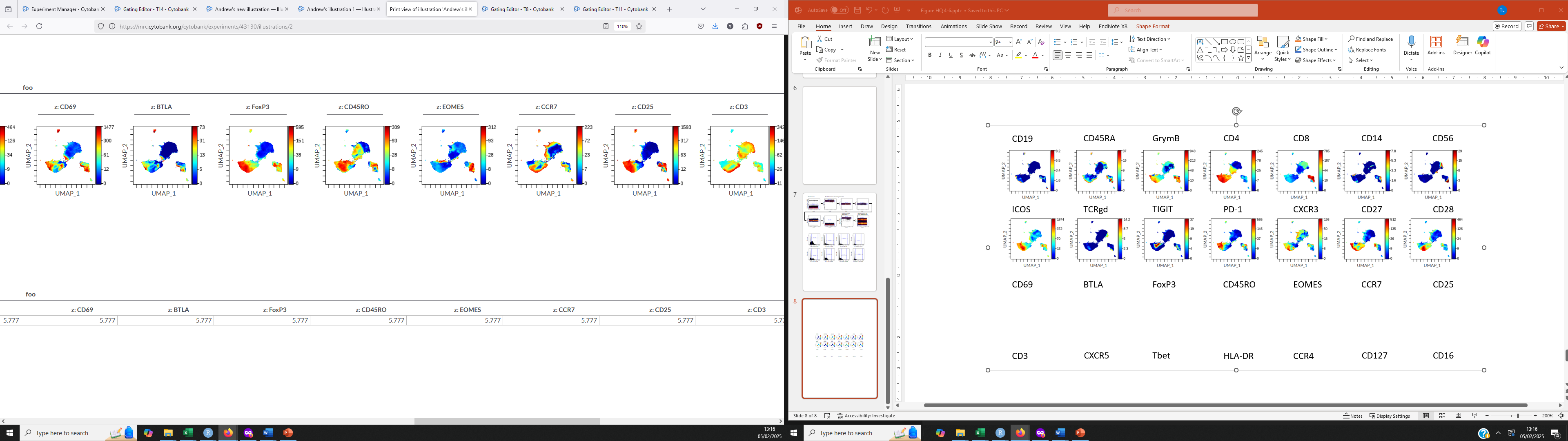


CD19

ICOS

CD45RA

GrymB

CD4

CD8

CD14

CD56

TIGIT

PD-1

CXCR3

CD27

CD28

CD69

BTLA

FoxP3

CD45RO

EOMES

CCR7

CD25

CD3

CXCR5

Tbet

HLA-DR

CCR4

CD127

CD16


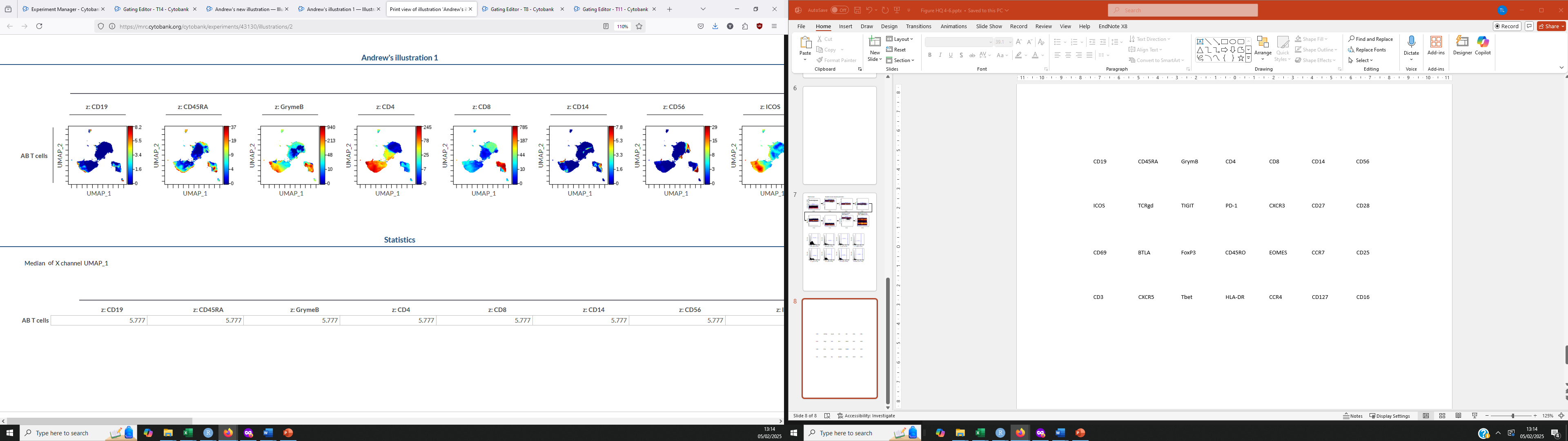

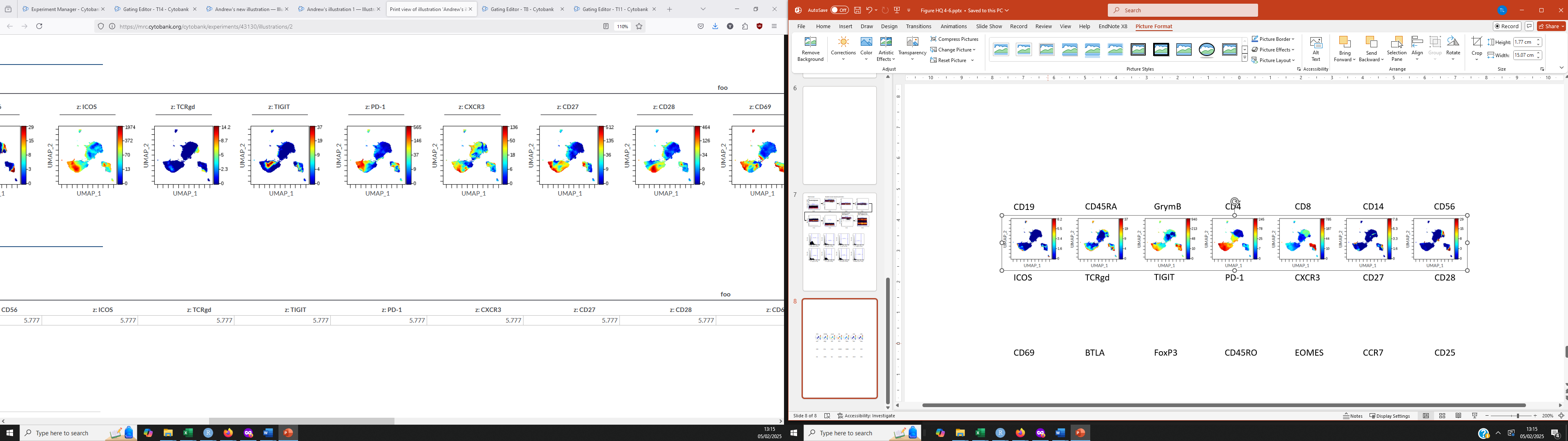

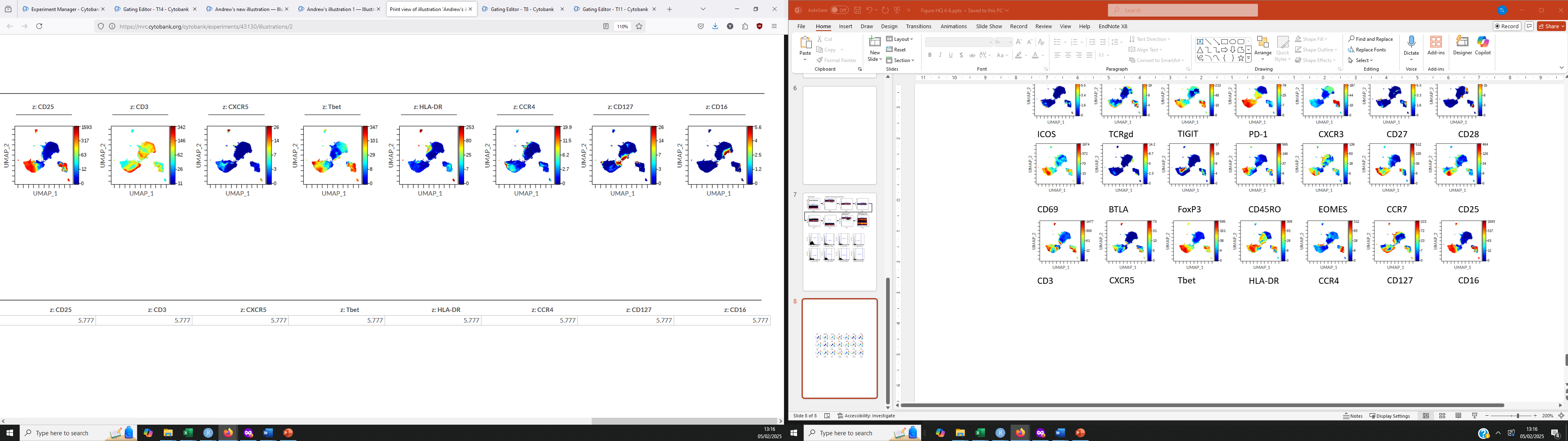


TCRgd

**Figure S5:** UMAPs representing ex-vivo CD3/CD28 stimulated αβ CD3^+^ T cells from patients with CLL showing the expression levels of extra- and intra-cellular markers.


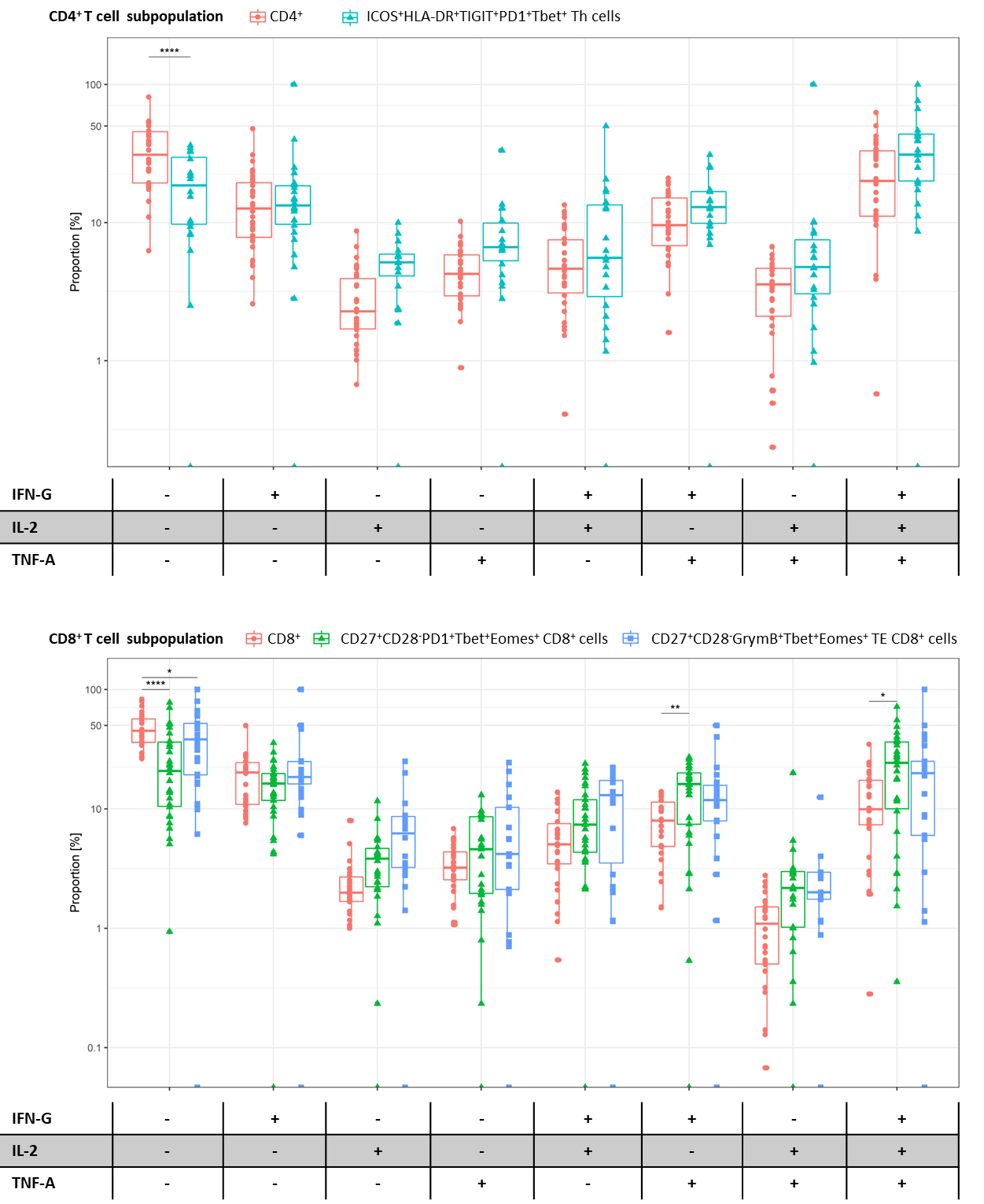


(B)

(A)

**Figure S6**: Individual patient analysis of pro-inflammatory cytokine expression in clinically significant T-cells populations in (A) CD4^+^ and (B) CD8^+^ T-cells. For each number of simultaneously expressed cytokines, results are compared between the parental CD4^+^ or CD8^+^ population and the subpopulation(s) of interest.

**Supplementary Tables:**

| **Marker** | **Clone** | **Isotope** | **Titration** | **Source (conjugation method)** |
| --- | --- | --- | --- | --- |
| CD45* | H130 | 89Yb | 1:100 | Biolegend, UK (IH) |
| CD45* | H130 | 106Cd | 1:100 | Biolegend, UK (IH) |
| CD45* | H130 | 110Cd | 1:100 | Biolegend, UK (IH) |
| CD45* | H130 | 111Cd | 1:100 | Biolegend, UK (IH) |
| CD45* | H130 | 113Cd | 1:100 | Biolegend, UK (IH) |
| CD45* | H130 | 114Cd | 1:100 | Biolegend, UK (IH) |
| CD45* | H130 | 115In | 1:100 | Biolegend, UK (IH) |
| CD45* | H130 | 116Cd | 1:100 | Biolegend, UK (IH) |
| CD196 (CCR6) | 11A9 | 141Pr | 1:100 | Standard Biotools, UK (P) |
| CD19 | HIB19 | 142Nd | 1:200 | Standard Biotools, UK (P) |
| CD45RA | HI100 | 143Nd | 1:50 | Biolegend, UK (IH) |
| CD4 | RPA-T4 | 145Nd | 1:200 | Standard Biotools, UK (P) |
| CD8a | RPA-T8 | 146Nd | 1:200 | Standard Biotools, UK (P) |
| CD123 | 6H6 | 147Sm | 1:100 | Biolegend, UK (IH) |
| CD14 | RMO52 | 148Nd | 1:100 | Standard Biotools, UK (P) |
| CD56 (NCAM) | NCAM16.2 | 149Sm | 1:100 | Standard Biotools, UK (P) |
| CD134 (OX40) | ACT35 | 150Nd | 1:100 | Standard Biotools, UK (P) |
| CD278/ICOS | C398.4A | 151Eu | 1:100 | Standard Biotools, UK (P) |
| TCRgd | 11F2 | 152Sm | 1:100 | Standard Biotools, UK (P) |
| TIM-3 | F38-2E2 | 153Eu | 1:100 | Standard Biotools, UK (P) |
| TIGIT | MBSA43 | 154Sm | 1:100 | Standard Biotools, UK (P) |
| CD279 (PD-1) | E12.2H7 | 155Gd | 1:100 | Standard Biotools, UK (P) |
| CD183 (CXCR3) | G025H7 | 156Gd | 1:100 | Standard Biotools, UK (P) |
| CD27 | L128 | 158Gd | 1:100 | Standard Biotools, UK (P) |
| CD223/LAG-3 | 11C3C65 | 159Tb | 1:100 | Biolegend, UK (IH) |
| CD28 | CD28.2 | 160Gd | 1:100 | Standard Biotools, UK (P) |
| CTLA-4 | 14D3 | 161Dy | 1:100 | Standard Biotools, UK (P) |
| CD69 | FN50 | 162Dy | 1:100 | Standard Biotools, UK (P) |
| BTLA | MIH26 | 163Dy | 1:100 | Standard Biotools, UK (P) |
| CD45RO | UCHL1 | 165Ho | 1:50 | Biolegend, UK (IH) |
| CD197 (CCR7) | G043H7 | 167Er | 1:100 | Standard Biotools, UK (P) |
| CD25 (IL-2R) | 2A3 | 169Tm | 1:100 | Standard Biotools, UK (P) |
| CD3 | UCHT1 | 170Er | 1:100 | Standard Biotools, UK (P) |
| CXCR5 | RF8B2 | 171Yb | 1:100 | Standard Biotools, UK (P) |
| CD137/4-1BB | 4B4-1 | 173Yb | 1:100 | Standard Biotools, UK (P) |
| HLA-DR | L243 | 174Yb | 1:100 | Standard Biotools, UK (P) |
| CCR4 | L291H4 | 175Lu | 1:100 | Standard Biotools, UK (P) |
| CD127 (IL-7R) | A019D5 | 176Yb | 1:100 | Standard Biotools, UK (P) |
| CD16 | 3G8 | 209Bi | 1:100 | Standard Biotools, UK (P) |
| Granzyme B^◊^ | CLB-GB11 | 144Nd | 1:400 | ThermoFisher, UK (IH) |
| FoxP3^◊^ | PCH101 | 164Dy | 1:50 | ThermoFisher, UK (IH) |
| Eomes^◊^ | WD1928 | 166Er | 1:50 | ThermoFisher, UK (IH) |
| TOX^◊^ | REA473 | 168Er | 1:50 | Miltenyi Biotec, UK (IH) |
| T-bet^◊^ | 4B10 | 172Yb | 1:50 | Biolegend, UK (IH) |

* Barcoding antibodies: eight-choose-two combinations were allocated for each sample
^◊^ Internal antibodies

**Table S1:** Markers included in CyTOF antibody panel for analysis of unstimulated primary samples. IH: in-house conjugation, P: purchased pre-conjugated.

| **Marker** | **Clone** | **Isotope** | **Titration** | **Source (conjugation method)** |
| --- | --- | --- | --- | --- |
| CD45* | H130 | 89Yb | 1:100 | Biolegend, UK (IH) |
| CD45* | H130 | 106Cd | 1:100 | Biolegend, UK (IH) |
| CD45* | H130 | 110Cd | 1:100 | Biolegend, UK (IH) |
| CD45* | H130 | 111Cd | 1:100 | Biolegend, UK (IH) |
| CD45* | H130 | 113Cd | 1:100 | Biolegend, UK (IH) |
| CD45* | H130 | 114Cd | 1:100 | Biolegend, UK (IH) |
| CD45* | H130 | 115In | 1:100 | Biolegend, UK (IH) |
| CD45* | H130 | 116Cd | 1:100 | Biolegend, UK (IH) |
| CD19 | HIB19 | 142Nd | 1:200 | Standard Biotools, UK (P) |
| CD45RA | HI100 | 143Nd | 1:50 | Biolegend, UK (IH) |
| CD4 | RPA-T4 | 145Nd | 1:200 | Standard Biotools, UK (P) |
| CD8a | RPA-T8 | 146Nd | 1:200 | Standard Biotools, UK (P) |
| CD14 | RMO52 | 148Nd | 1:100 | Standard Biotools, UK (P) |
| CD56 (NCAM) | NCAM16.2 | 149Sm | 1:100 | Standard Biotools, UK (P) |
| CD278/ICOS | C398.4A | 151Eu | 1:100 | Standard Biotools, UK (P) |
| TCRgd | 11F2 | 152Sm | 1:100 | Standard Biotools, UK (P) |
| TIGIT | MBSA43 | 154Sm | 1:100 | Standard Biotools, UK (P) |
| CD279 (PD-1) | E12.2H7 | 155Gd | 1:100 | Standard Biotools, UK (P) |
| CD183 (CXCR3) | G025H7 | 156Gd | 1:100 | Standard Biotools, UK (P) |
| CD27 | L128 | 158Gd | 1:100 | Standard Biotools, UK (P) |
| CD28 | CD28.2 | 160Gd | 1:100 | Standard Biotools, UK (P) |
| CD69 | FN50 | 162Dy | 1:100 | Standard Biotools, UK (P) |
| BTLA | MIH26 | 163Dy | 1:100 | Standard Biotools, UK (P) |
| CD45RO | UCHL1 | 165Ho | 1:50 | Biolegend, UK (IH) |
| CD197 (CCR7) | G043H7 | 167Er | 1:100 | Standard Biotools, UK (P) |
| CD25 (IL-2R) | 2A3 | 169Tm | 1:100 | Standard Biotools, UK (P) |
| CD3 | UCHT1 | 170Er | 1:100 | Standard Biotools, UK (P) |
| CXCR5 | RF8B2 | 171Yb | 1:100 | Standard Biotools, UK (P) |
| HLA-DR | L243 | 174Yb | 1:100 | Standard Biotools, UK (P) |
| CD127 (IL-7R) | A019D5 | 176Yb | 1:100 | Standard Biotools, UK (P) |
| CD16 | 3G8 | 209Bi | 1:100 | Standard Biotools, UK (P) |
| IL-2^◊^ | MQ1-17H12 | 141Pr | 1:50 | Biolegend, UK (IH) |
| Granzyme B^◊^ | CLB-GB11 | 144Nd | 1:400 | ThermoFisher, UK (IH) |
| IFN-γ^◊^ | B27 | 147Sm | 1:50 | Biolegend, UK (IH) |
| IL-17A^◊^ | BL168 | 150Nd | 1:50 | Biolegend, UK (IH) |
| TNF-α^◊^ | MAb11 | 153Eu | 1:50 | Biolegend, UK (IH) |
| IL-10^◊^ | JES3-9D7 | 159Tb | 1:50 | Biolegend, UK (IH) |
| IL-4^◊^ | MP4-25D2 | 161Dy | 1:50 | Biolegend, UK (IH) |
| FoxP3^◊^ | PCH101 | 164Dy | 1:50 | ThermoFisher, UK (IH) |
| Eomes^◊^ | WD1928 | 166Er | 1:50 | ThermoFisher, UK (IH) |
| TGF-β^◊^ | TGF-β1 | 168Er | 1:100 | Biolegend, UK (IH) |
| T-bet^◊^ | 4B10 | 172Yb | 1:50 | Biolegend, UK (IH) |
| Perforin^◊^ | dG9 | 173Yb | 1:100 | Biolegend, UK (IH) |

* Barcoding antibodies: eight-choose-two combinations were allocated for each sample
^◊^ Internal antibodies

**Table S2:** Markers included in CyTOF antibody panel for analysis of *ex vivo* CD3/CD28 stimulated primary samples. IH: in-house conjugation, P: purchased pre-conjugated.


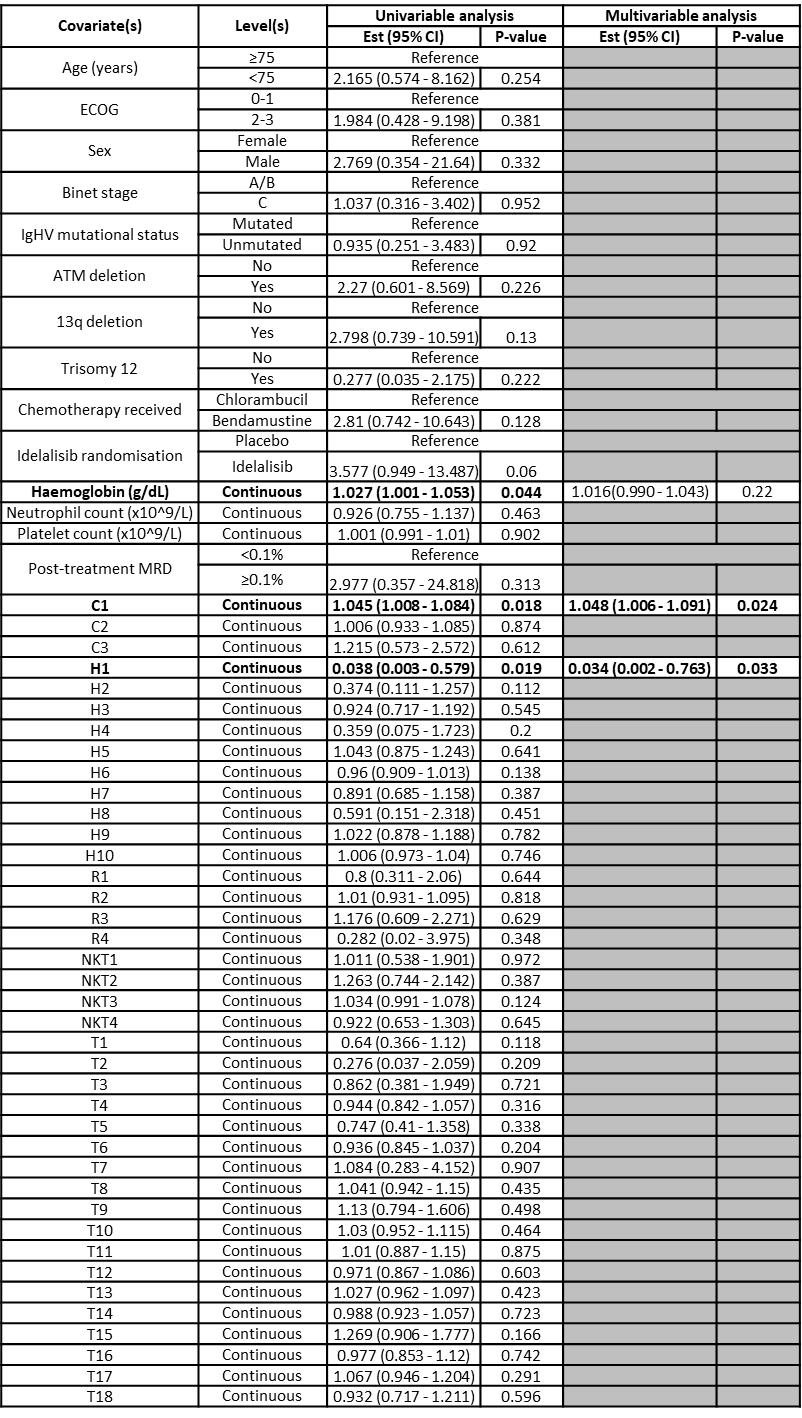


**Table S3:** Univariable and multivariable analyses of subsequent risk of grade ≥3 infections, with estimated effect (Est), 95% confidence interval (CI) and statistical significance for all potential covariates. ECOG: Eastern Cooperative Oncology Group, IgHV: Immunoglobin heavy chain variable, MRD: measurable residual disease.


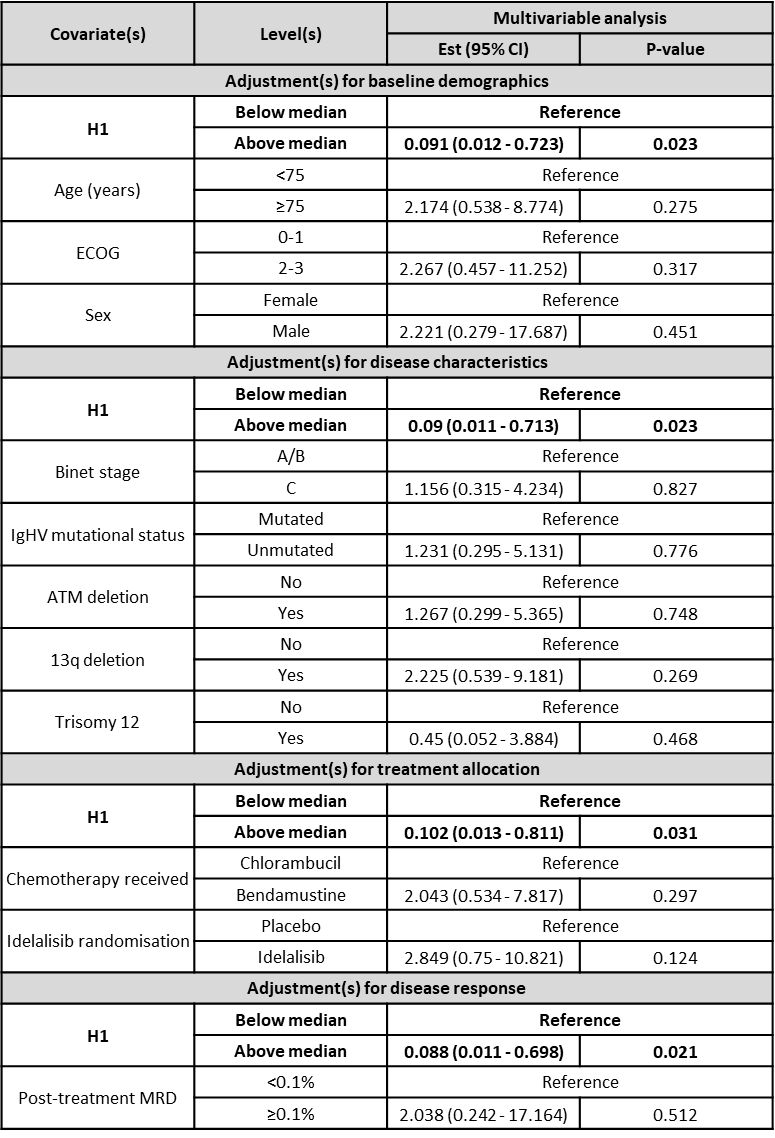


**Table S4:** Sensitivity analysis of the association between H1 cluster frequency and risk of severe infections, statistically adjusted for baseline demographics, disease characteristics, treatment allocation, and disease response


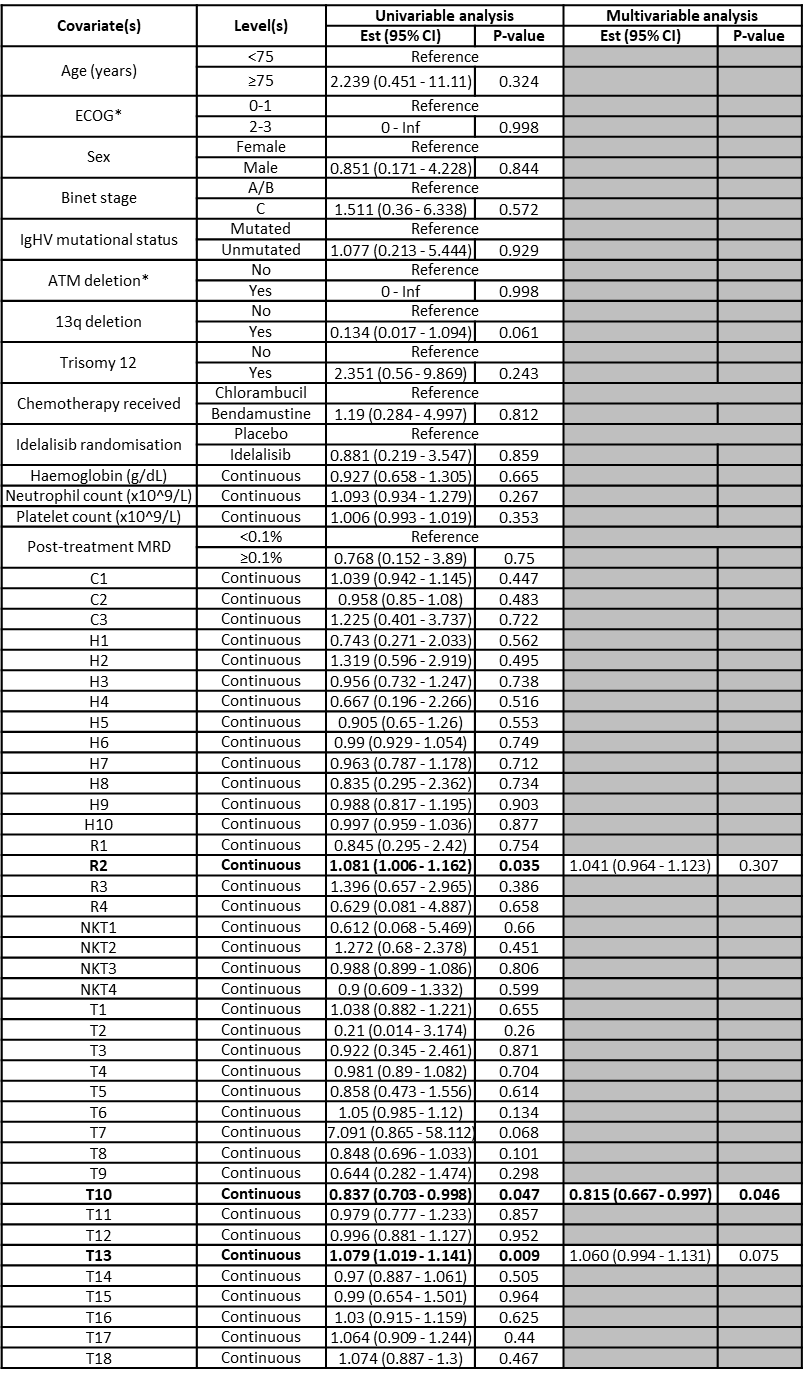
**Table S5:** Univariable and multivariable analyses of subsequent risk of grade ≥3 secondary primary malignancies (SPMs), with estimated effect (Est), 95% confidence interval (CI) and statistical significance for all potential covariates. ECOG: Eastern Cooperative Oncology Group, IgHV: Immunoglobin heavy chain variable, MRD: measurable residual disease. *There were no SPM events in the comparing group.


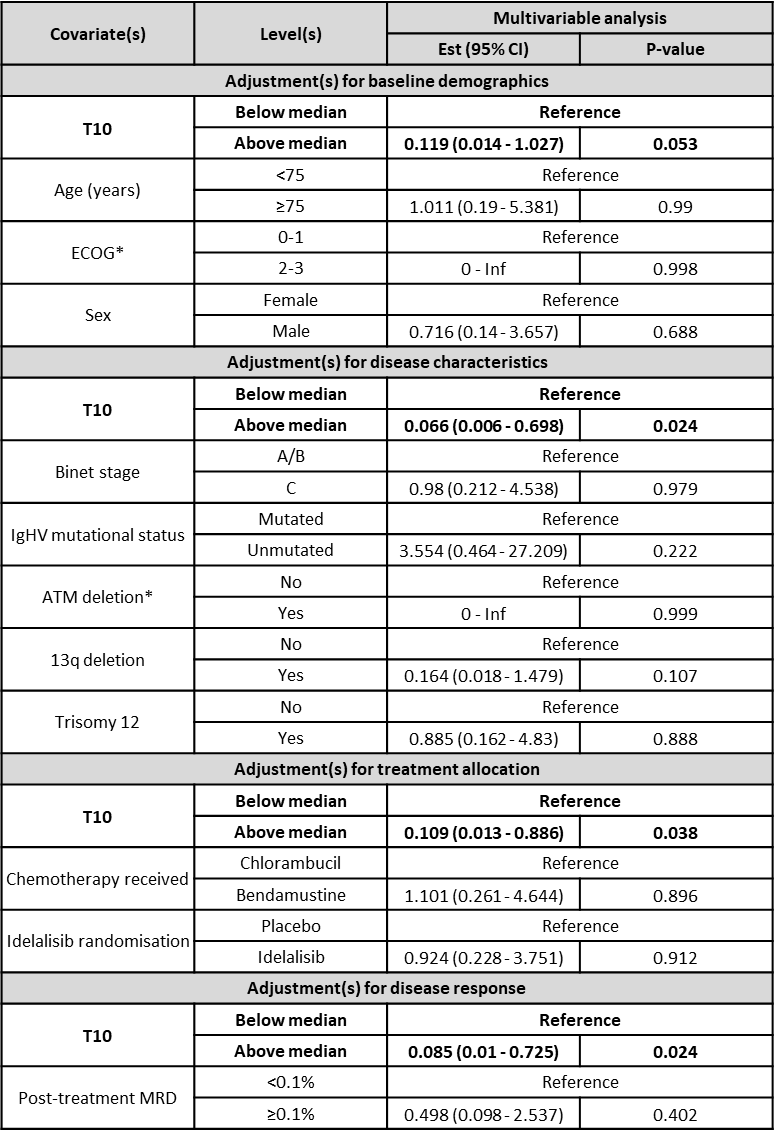


**Table S6:** Sensitivity analysis of the association between T10 cluster frequency and risk of SPMs, statistically adjusted for baseline demographics, disease characteristics, treatment allocation, and disease response. *There were no SPM events in the comparing group.


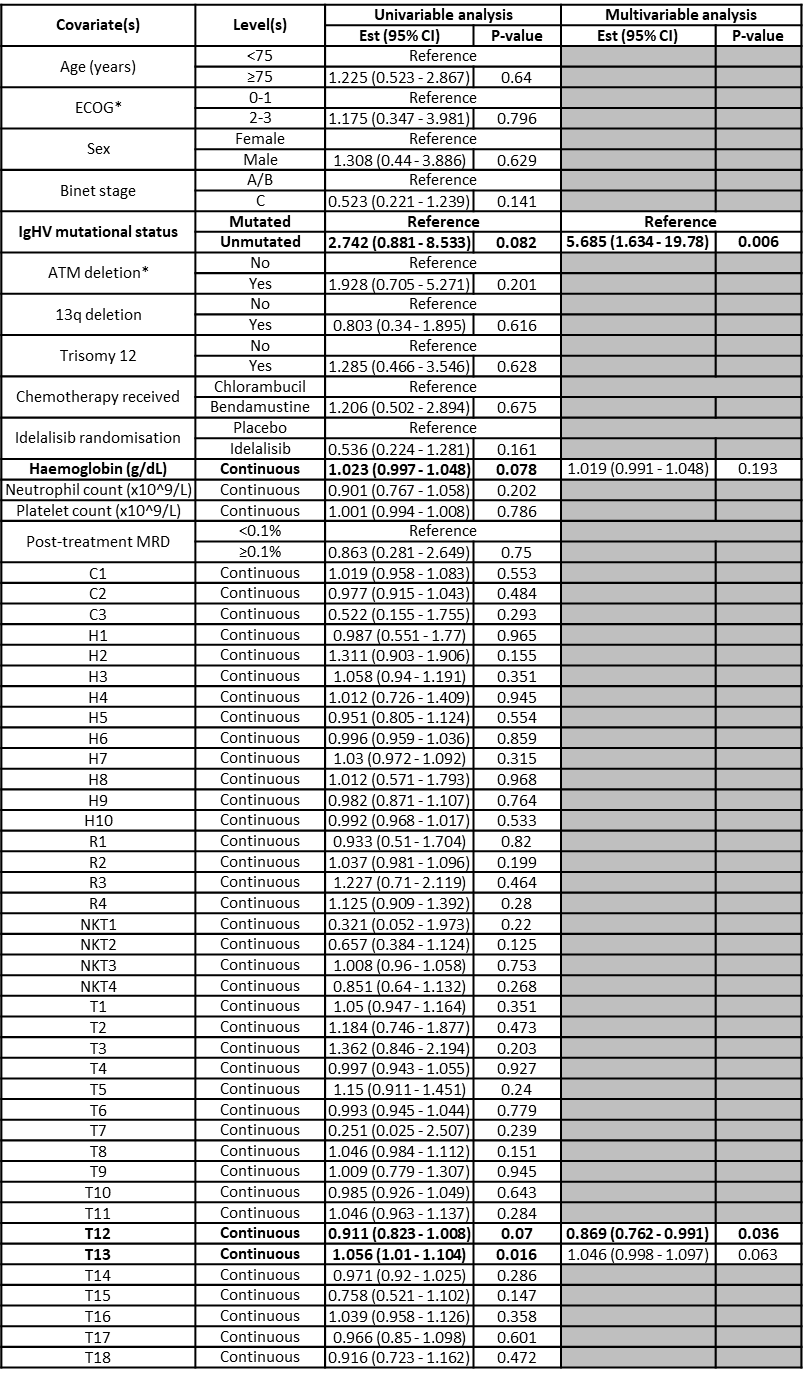


**Table S7:** Univariable and multivariable analyses of overall survival (OS), with estimated effect (Est), 95% confidence interval (CI) and statistical significance for all potential covariates. ECOG: Eastern Cooperative Oncology Group, IgHV: Immunoglobin heavy chain variable, MRD: measurable residual disease.


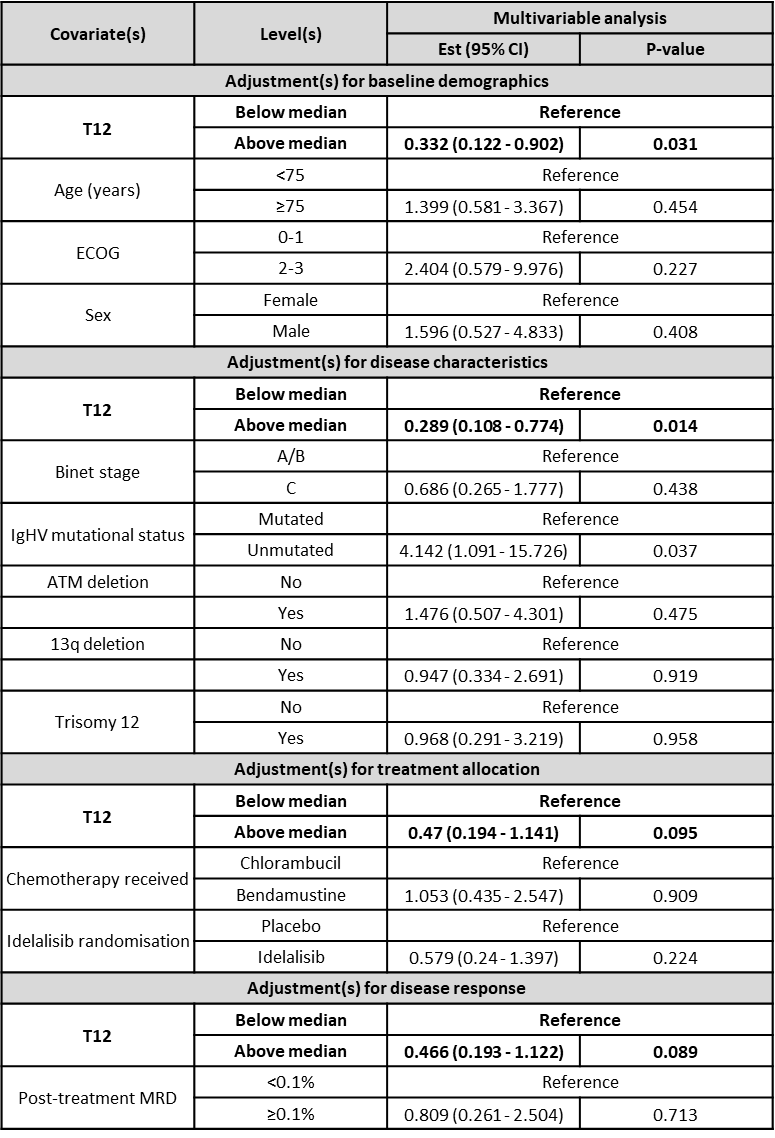


**Table S8:** Sensitivity analysis of the association between T12 cluster frequency and OS, statistically adjusted for baseline demographics, disease characteristics, treatment allocation, and disease response

| **EoT response** | **Significant cluster** | **Est (95% CI)** | **Z value** | **P-value** | |
| --- | --- | --- | --- | --- | --- |
|  |  |  |  | **Univariable** | **Multivariable** |
| MRD2  (1: >1%, 0: <1%) | **H6** | **0.523 (0.318 - 0.728)** | **2.554** | **0.011** | **n/a*** |
| MRD3  (1: >0.1%, 0: <0.1%) | **NKT3** | **0.145 (0.086 - 0.204)** | **2.469** | **0.014** | **0.039** |
|  | T4 | -0.115 (-0.17 - -0.06) | -2.08 | 0.037 | 0.199 |
| MRD4  (1: >0.01%, 0: <0.01%) | R2 | -0.113 (-0.166−-0.06) | -2.128 | 0.033 | 0.117 |
|  | **T1** | **-0.262 (-0.363−-0.161)** | **-2.603** | **0.009** | **0.0377** |

**Table S9:** Univariable and multivariable logistic regression analysis showing FlowSOM clusters that correlate with EoT MRD. Statistically significant associations on multivariable analysis are highlighted in bold.*H6 was the only significant cluster on univariable analysis.

| **Variables** | **HR (95% CI)** | **z value** | **P value** |
| --- | --- | --- | --- |
| R1 | 0.944 (0.63 - 1.415) | -0.279 | 0.780 |
| R2 | 1.007 (0.966 - 1.05) | 0.323 | 0.747 |
| R3 | 1.214 (0.862 - 1.71) | 1.112 | 0.266 |
| H1 | 0.845 (0.568 - 1.257) | -0.833 | 0.405 |
| H2 | 0.915 (0.622 - 1.348) | -0.448 | 0.654 |
| H3 | 0.984 (0.89 - 1.087) | -0.324 | 0.746 |
| H4 | 1.066 (0.865 - 1.313) | 0.601 | 0.548 |
| H5 | 1.088 (0.865 - 1.368) | 0.721 | 0.471 |
| H6 | 1.023 (0.946 - 1.106) | 0.568 | 0.570 |
| H7 | 1.022 (0.996 - 1.049) | 1.686 | 0.092 |
| H8 | 1.015 (0.975 - 1.056) | 0.729 | 0.466 |
| H9 | 1.009 (0.724 - 1.407) | 0.053 | 0.958 |
| H10 | 1.013 (0.944 - 1.087) | 0.350 | 0.727 |
| H11 | 0.992 (0.976 - 1.009) | -0.892 | 0.372 |
| C1 | 0.99 (0.933 - 1.049) | -0.352 | 0.725 |
| C2 | 0.975 (0.936 - 1.016) | -1.184 | 0.237 |
| C3 | 1.141 (0.716 - 1.816) | 0.554 | 0.579 |
| NKT1 | 1.075 (0.839 - 1.378) | 0.571 | 0.568 |
| NKT2 | 1.068 (0.82 - 1.392) | 0.488 | 0.626 |
| NKT3 | 1.021 (0.987 - 1.055) | 1.202 | 0.229 |
| NKT4 | 0.897 (0.77 - 1.045) | -1.393 | 0.164 |
| T1 | 0.965 (0.881 - 1.057) | -0.767 | 0.443 |
| T2 | 0.945 (0.631 - 1.415) | -0.275 | 0.783 |
| T3 | 0.904 (0.596 - 1.371) | -0.475 | 0.635 |
| T4 | 0.98 (0.942 - 1.02) | -0.975 | 0.330 |
| T5 | 0.986 (0.801 - 1.213) | -0.133 | 0.894 |
| T6 | 0.992 (0.956 - 1.029) | -0.447 | 0.655 |
| T7 | 2.627 (1.179 - 5.856) | 2.362 | 0.018* |
| T8 | 0.976 (0.925 - 1.031) | -0.860 | 0.390 |
| T9 | 0.854 (0.695 - 1.05) | -1.498 | 0.134 |
| T10 | 1.012 (0.971 - 1.054) | 0.550 | 0.582 |
| T11 | 1.043 (0.975 - 1.116) | 1.215 | 0.224 |
| T12 | 0.989 (0.944 - 1.036) | -0.465 | 0.642 |
| T13 | 1.007 (0.965 - 1.05) | 0.311 | 0.756 |
| T14 | 1.005 (0.977 - 1.034) | 0.319 | 0.749 |
| T15 | 0.994 (0.839 - 1.178) | -0.070 | 0.945 |
| T16 | 1.038 (0.992 - 1.085) | 1.604 | 0.109 |
| T17 | 0.977 (0.901 - 1.059) | -0.574 | 0.566 |
| T18 | 1.027 (0.937 - 1.127) | 0.572 | 0.567 |

**Table S10:** Univariable coxph regression analysis of cluster frequencies and time to progression (TTP). *Despite a significant P-value on coxph analysis, K-M analysis of patients stratified based on the median T7 cluster size showed no significant difference in TTP.
